# Humanized *GPRC6A*^*KGKY*^ is a gain-of-function polymorphism in mice

**DOI:** 10.1038/s41598-020-68113-z

**Published:** 2020-07-07

**Authors:** Min Pi, Fuyi Xu, Ruisong Ye, Satoru K. Nishimoto, Robert A. Kesterson, Robert W. Williams, Lu Lu, L. Darryl Quarles

**Affiliations:** 1 Department of Medicine, University of Tennessee Health Science Center, 19 S Manassas St., Memphis, TN 38163 USA; 2 Department of Genetics, Genomics and Informatics, University of Tennessee Health Science Center, 19 S Manassas St., Memphis, TN 38163 USA; 3 Department of Microbiology, Immunology and Biochemistry, University of Tennessee Health Science Center, 19 S Manassas St., Memphis, TN 38163 USA; 4 Department of Genetics, University of Alabama at Birmingham, 720 20th Street South, Birmingham, AL 35294 USA

**Keywords:** Molecular biology, Endocrinology

## Abstract

GPRC6A is proposed to regulate energy metabolism in mice, but in humans a KGKY polymorphism in the third intracellular loop (ICL3) is proposed to result in intracellular retention and loss-of-function. To test physiological importance of this human polymorphism in vivo, we performed targeted genomic humanization of mice by using CRISPR/Cas9 (clustered regularly interspaced short palindromic repeats-CRISPR associated protein 9) system to replace the RKLP sequence in the ICL3 of the GPRC6A mouse gene with the uniquely human KGKY sequence to create *Gprc6a-*^*KGKY-knockin*^ mice. Knock-in of a human KGKY sequence resulted in a reduction in basal blood glucose levels and increased circulating serum insulin and FGF-21 concentrations. *Gprc6a-*^*KGKY-knockin*^ mice demonstrated improved glucose tolerance, despite impaired insulin sensitivity and enhanced pyruvate-mediated gluconeogenesis. Liver transcriptome analysis of *Gprc6a-*^*KGKY-knockin*^ mice identified alterations in glucose, glycogen and fat metabolism pathways. Thus, the uniquely human *GPRC6A-*^*KGKY*^ variant appears to be a gain-of-function polymorphism that positively regulates energy metabolism in mice.

## Introduction

GPRC6A is a family C G-protein coupled receptor (GPCR) that is reported to be capable of sensing multiple ligands. There is a consensus that GPRC6A is activated by basic amino acid, including l-arginine, l-ornithine, and l-lysine^1–3^. Several laboratories have shown that GPRC6A is activated by the bone-derived peptide, osteocalcin (Ocn)^4–10^. and testosterone (T)^1,11–13^, and natural products in green tea^14^. In contrast, other investigators have failed to show that either testosterone or Ocn activates GPRC6A^15,16^. GPRC6A is expressed in several tissues, including β-cells, liver hepatocytes, skeletal muscle, fat, and Leydig cells where it is reported to regulate glucose and fat metabolism and hormone production.

*Gprc6a*^*−/−*^ mice exhibit complex metabolic derangements that resemble metabolic syndrome (MetS), including glucose intolerance, insulin resistance, and fatty liver^1,9,17–20^. Conditional deletion of *Gprc6a* in Leydig cells attenuates Ocn induced T production by the testes^21^*,* in pancreatic β-cells regulates insulin secretion^9,19^, and in skeletal muscle regulates muscle glucose and fatty acid utilization and IL-6 production independent of insulin during exercise^20,22,23^. The similar phenotypes of *Gprc6a*^*−/−*^ and *Ocn*^*−/−*^ mice and the additive phenotypic effects in compound *Gprc6a*^+*/−*^*/Ocn*^+*/−*^ mice support the physiological importance of Ocn as the cognate GPRC6A ligand^24^. The GPRC6A endocrine network appears to be strongly influenced by genetic, sex-dependent and environmental factors, since the phenotype of Ocn and GPRC6A deficiencies are variable^3,15,16,24–28^.

The clinical significance of GPRC6A in regulating energy metabolism in humans remains to be established. The ancestral RKLP polymorphism in the 3rd intracellular loop found in mice and all other species is a minor variant in humans, most commonly found in African Americans (40%)^15,16,28,29^. Rather a *GPRC6A-*^*KGKY*^ variant, which replaces the RKLP sequence in the 3rd intracellular loop with the KGKY insertion/deletion, evolved uniquely in humans^25,30^ and is present in 91% of Europeans, and 99% of Asians, but only 60% of people of African descent. While the ancestral *GPRC6A-*^*RKLP*^ variant is located on the cell surface and undergoes ligand-dependent recycling like classical G-protein coupled receptors, the newly evolved *GPRC6A-*^*KGKY*^ variant is predominantly located intracellularly in the early endosomes^13,26^. The *GPRC6A-*^*KGKY*^ variant is inconsistently reported to be either a loss-of-function^26,31^, or a gain-of-function polymorphism^13,32^ using in vitro cell culture model systems.

To understand the function of this uniquely human KGKY polymorphism in vivo, we have knocked-in the KGKY sequence to replace the ancestral RKLP sequence in the 3rd intracellular loop of the mouse gene. The transgenic mouse model establishes the KGKY insertion/deletion as a gain-of-function polymorphism in vivo.

## Results

### “Humanized” *Gprc6a-*^*KGKY-knockin*^ transgenic mice have enhanced metabolic functions

Our previous data found that the insertion of the KGKY sequence in the mouse GPRC6A (i.e., humanized mouse GPRC6A^ICL3_KGKY^) acquires characteristics of the human GPRC6A^ICL3_KGKY^, namely predominate location to endosome-like intracellular punctuate structures and gain-of-function of mTOR signaling, an evolutionarily conserved pathway in endosomal nutrient signaling^13,32^. We extend these data, by showing that osteocalcin induces an increase in the magnitude and duration of mTOR signaling in cells transfected with the mGPRC6A^ICL3_KGKY^ mutant compared to the wild-type mouse GPRC6A^ICL3_RKLP^ cDNA in vitro (Figure S1A). In contrast, activation of ERK signaling by osteocalcin was not different between transfected mGPRC6A^ICL3_KGKY^ mutant compared to the wild-type GPRC6A^ICL3_RKLP^ (Figure S1B).

**Figure 1:**
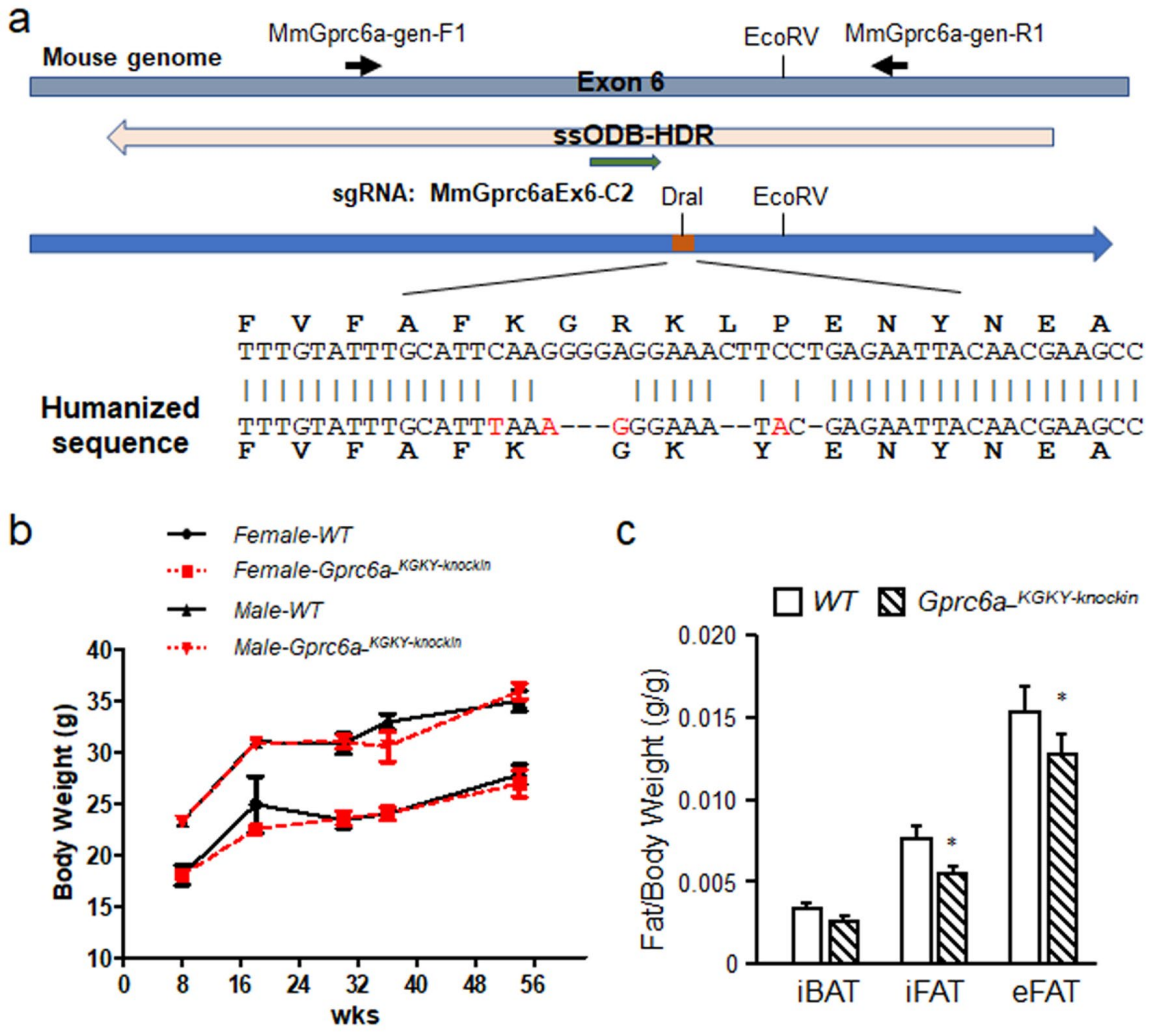
Creation of *Gprc6a-*^*KGKY-knockin*^ mice. (**a**) Structure of *Gprc6a* exon 6, knockin sequence and genotype primers and *Dra* I restricted enzyme location. The orange box shows the KY location in blue arrow. The yellow arrow shows the ssOBD-HDR location. The green arrow shows sgRNA location. (**b**) Comparison of the body weight in wild type and *Gprc6a-*^*KGKY-knockin*^ mice at age from 8 to 54 weeks. (**c**) Site specific brown and white fat content in 20 week-old male mice. Interscapular brown fat (iBAT) and white inguinal fat (iFAT) and epididymal fat (eFAT). Values represent the mean ± SEM. *Significant difference between wild type mouse *Gprc6a-*^*RKLP*^ and *Gprc6a-*^*KGKY-knockin*^ mice (*P* < 0.05, Student’s *t* test; n = 6).

To test the function of this insertion/deletion in vivo, we “humanized” the mouse *Gprc6a* gene by using CRISPR/Cas9 system to replace the ICL3_RKLP sequence in the mouse with the ICL3_KGKY sequence (Fig. 1a). *Gprc6a-*^*KGKY-knockin*^ mice had similar body weights as *Gprc6a-*^*RKLP*^ wild type controls (Fig. 1b). *Gprc6a-*^*KGKY-knockin*^ male mice had significantly lower inguinal and epididymal white fat mass, but no change in intrascapular brown fat in 20 week-old of age (Fig. 1c).

**Figure 2:**
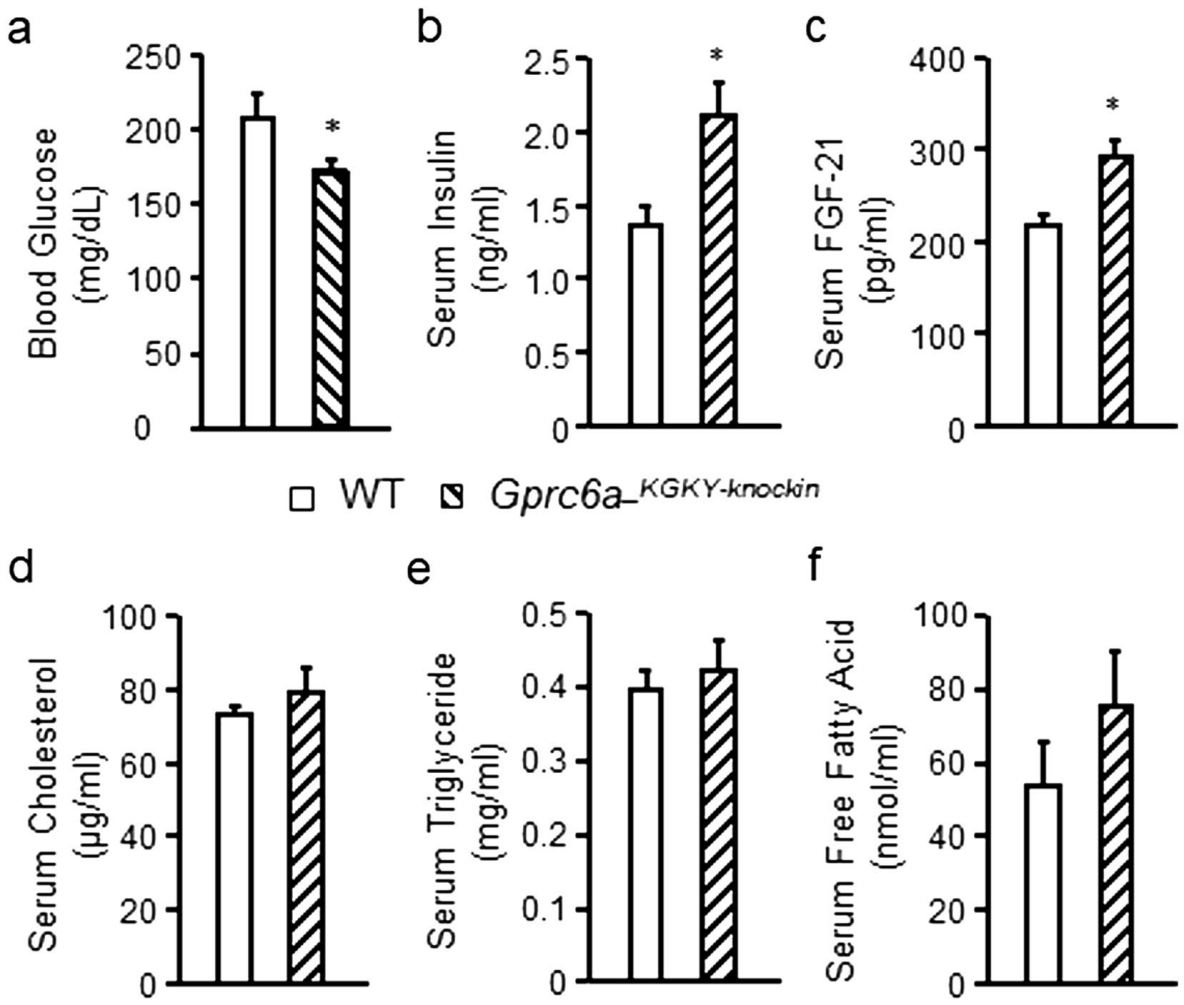
Characterization of *Gprc6a-*^*KGKY-knockin*^ mice. Comparison of the fasting blood glucose (**a**), serum insulin (**b**) and serum FGF-21 levels (**c**), serum levels of cholesterol (**d**), triglyceride (**e**) and free fatty acid (**f**) in wild type and *Gprc6a-*^*KGKY-knokin*^ male mice at age of 10 week-old. Values represent the mean ± SEM. *Significant difference between wild type and *Gprc6a-*^*KGKY-knockin*^ mice (*P* < 0.05, Student’s *t* test; n = 6).

*Gprc6a-*^*KGKY-knockin*^ mice had significantly lower fasting blood glucose concentrations (*P* ≤ 0.05, Fig. 2a) and a significant increase in basal serum insulin levels (*P* < 0.05; Fig. 2b). *Gprc6a-*^*KGKY-knockin*^ mice also exhibited significantly increased serum FGF-21 levels (*P* < 0.05, Fig. 2c). We found no significant changes in serum cholesterol, triglycerides or free fatty acid concentrations in the serum of *Gprc6a-*^*KGKY-knockin*^ mice compared to controls (Fig. 2d–f).

**Figure 3:**
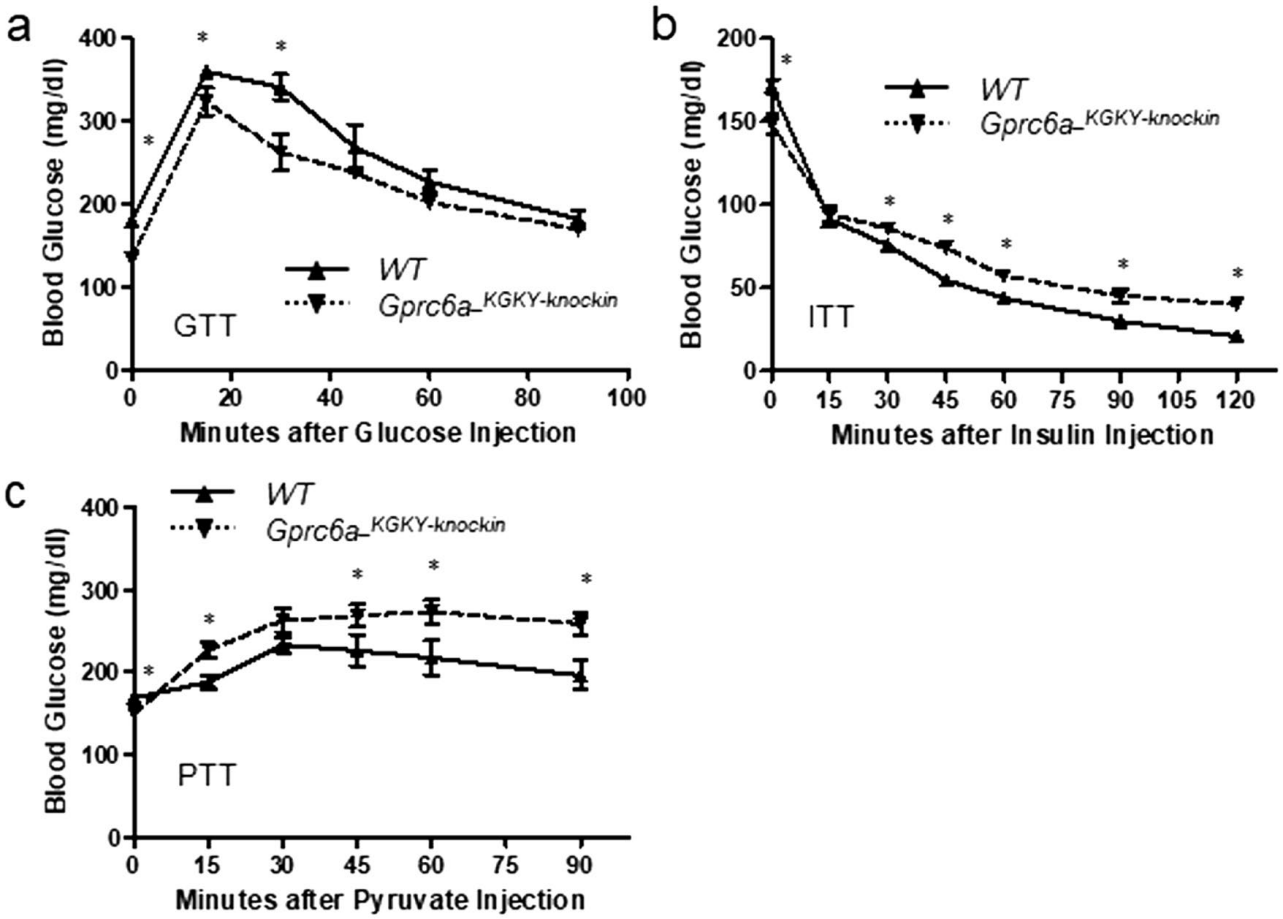
Tolerance tests in G*prc6a-*^*KGKY-knockin*^ mice. Blood glucose (mg/ml) during GTT (**a**), ITT (**b**) and PTT (**c**) in 10-week-old wild type and *Gprc6a-*^*KGKY-knockin*^ mice. Data represent the mean ± SEM from more than 5 male mice in each group. *Difference from wild type and *Gprc6a-*^*KGKY-knockin*^ mice at *P* < 0.05, two-way ANOVA with Tukey’s multiple comparisons test.

*Gprc6a-*^*KGKY-knockin*^ mice also had an improved glucose tolerance test (GTT) as shown by lower blood glucose concentrations compared to controls at different time points after glucose injection (Fig. 3a). Accordingly, the net area under the curve, which represents the variation in glucose concentration from baseline over the test duration, was smaller in *Gprc6a-*^*KGKY-knockin*^ mice.

*Gprc6a-*^*KGKY-knockin*^ mice lowered blood glucose in response to insulin, but they maintained a higher blood glucose compared to controls throughout most of the insulin tolerance test^33^ (Fig. 3b). The pyruvate tolerance test (PTT) assesses the effects of glucose production through gluconeogenesis. *Gprc6a-*^*KGKY-knockin*^ mice had a greater increase in blood glucose in response to pyruvate (Fig. 3c) compared to wild-type (*Gprc6a-*^*RKLP*^) mice, consistent with higher gluconeogenesis in *Gprc6a-*^*KGKY-knockin*^ mice.

**Figure 4:**
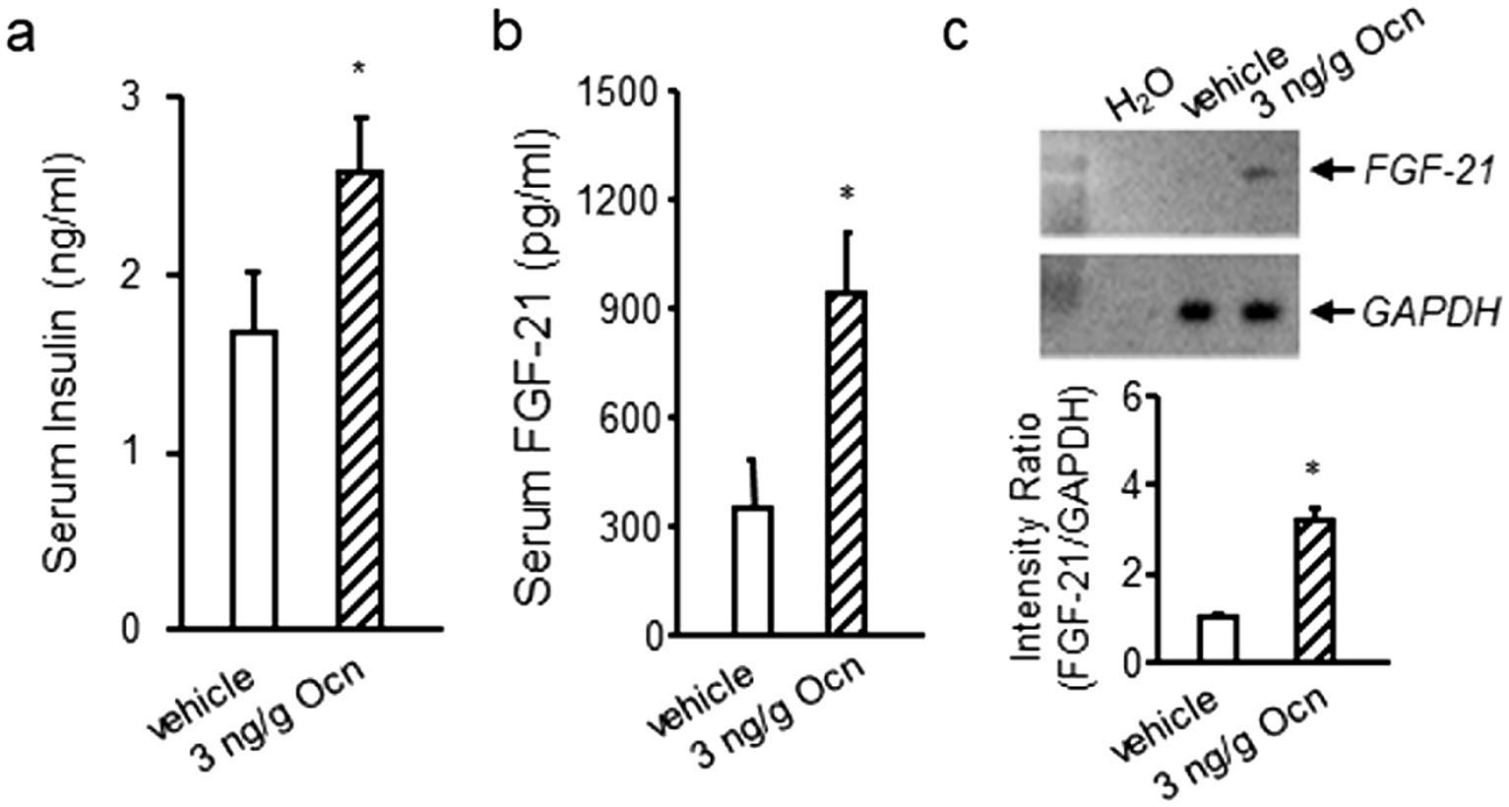
Effects of Ocn administration on insulin and FGF-21 in wild-type mice. Effect of Ocn administration to increase serum insulin (**a**) and FGF-21 (**b**) concentrations and liver message levels (**c**) in wild-type male mice. Values represent the mean ± SEM. *Significant difference between control group and Ocn-treated mice (*P* < 0.05, Student’s *t* test; n = 5).

Next, we compared the serum insulin and FGF-21 response in *Gprc6a-*^*KGKY-knockin*^ mice with the pharmacological effects of GPRC6A activation by its ligand Ocn. We found that Ocn administration resulted in increases in both insulin and FGF-21 circulating levels (Fig. 4a, b). In addition, Ocn stimulated FGF-21 message levels in the liver (Fig. 4c).

**Figure 5:**
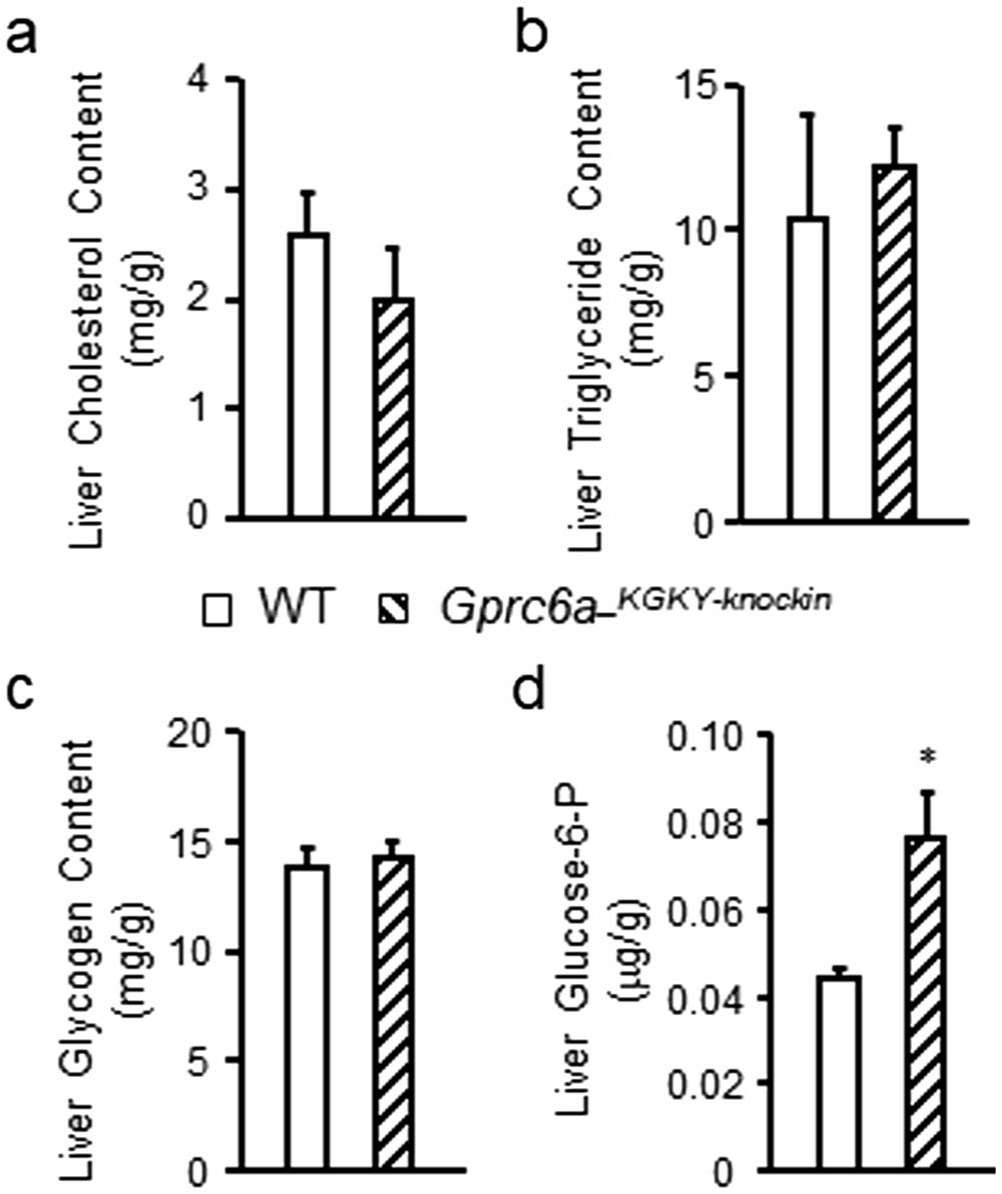
Characterization of additional liver parameters in *Gprc6a-*^*KGKY-knockin*^ mice. Comparison of the contents of cholesterol (**a**), triglyceride (**b**) and glycogen (**c**), and glucose-6-phosphate (Glucose-6-P) (**d**) in liver from control group and *Gprc6a-*^*KGKY-knokin*^ male mice at age of 10 week-old. Values represent the mean ± SEM. *Significant difference between control group and *Gprc6a-*^*KGKY-knockin*^ mice (*P* < 0.05, Student’s *t* test; n = 6).

Since FGF-21 is produced by the liver, we next examined additional hepatic parameters in *Gprc6a-*^*KGKY-knockin*^ mice. We found slight reductions in liver cholesterol content but no differences in liver triglyceride or glycogen content in *Gprc6a-*^*KGKY-knockin*^ mice (Fig. 5a–c). Liver glucose-6-P levels were significantly elevated in in *Gprc6a-*^*KGKY-knockin*^ mice compared to wild-type mice (Fig. 5d), consistent with GPRC6A regulating glucose metabolism in the liver.

### Liver transcriptome in *Gprc6a-*^*KGKY-knockin*^ mice

**Figure 6:**
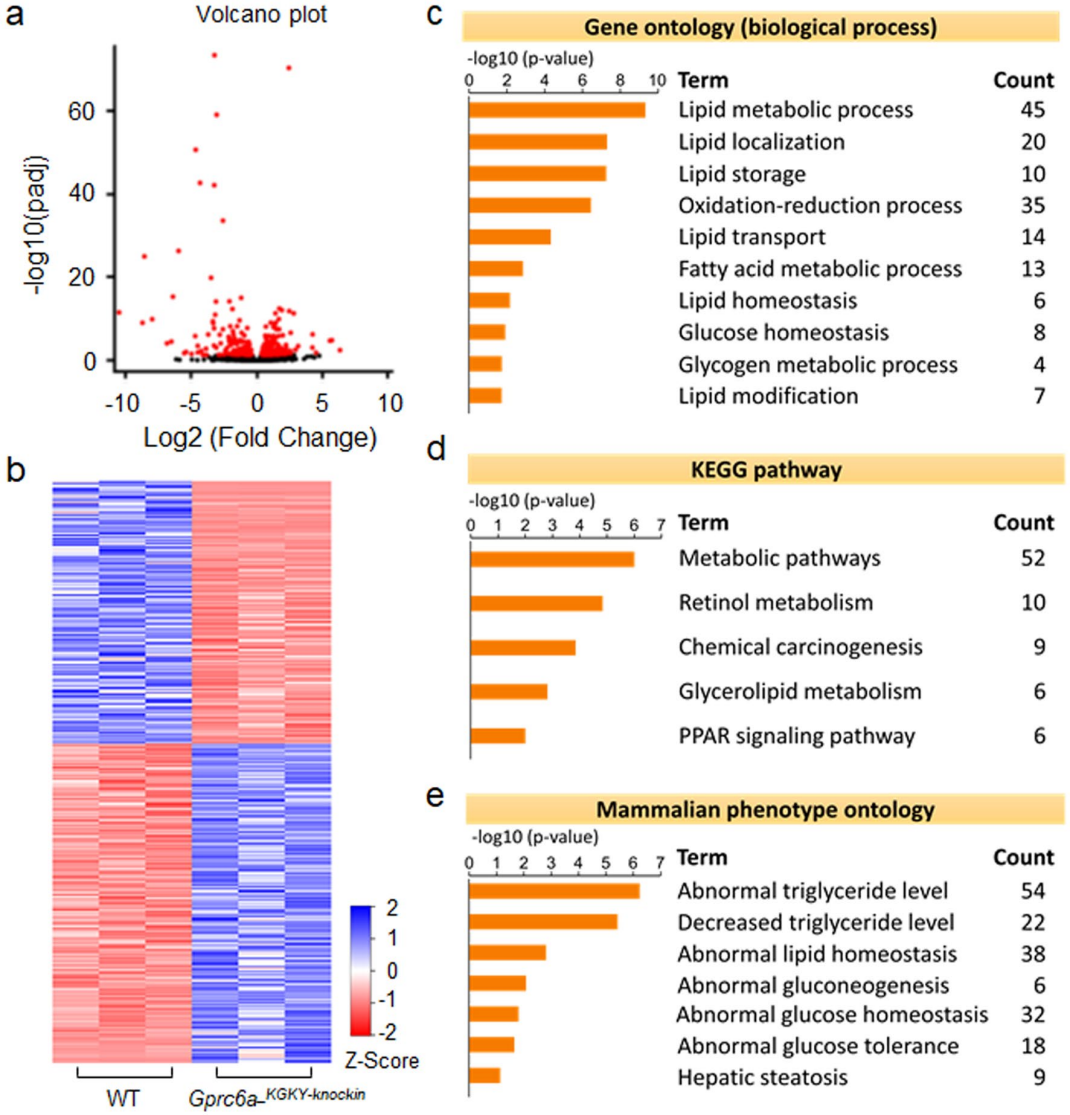
Hepatic gene expression in the liver of *Gprc6a-*^*KGKY-knockin*^ mice. (A and B) Volcano plot (**a**); Heat map (**b**) of regulated genes between WT and *Gprc6a-*^*KGKY-knockin*^ mice. Blue and red colors indicate high and low gene expression, respectively. Volcano plot and heat map visualization of the hepatic transcriptome demonstrated distinct differences between WT and *Gprc6a-*^*KGKY-knockin*^ mice. (**c**–**e**) Gene ontology (**c**), the Kyoto Encyclopedia of Genes and Genomes pathway (KEGG) (**d**) and Mammalian phenotype (**e**) in the liver of *Gprc6a-*^*KGKY-knockin*^ mice. The top rank ordered processes, maps and networks are based on statistical significance.

Finally, we examined the liver transcriptome in *Gprc6a-*^*KGKY-knockin*^ mice (Fig. 6). Volcano plot (Fig. 6a) and heat map visualization (Fig. 6b) of the hepatic transcriptome demonstrated distinct differences between wild-type and *Gprc6a-*^*KGKY-knockin*^ mice. A total of 359 (197 upregulated and 162 downregulated) genes were identified to be differentially expressed in livers from *Gprc6a-*^*KGKY-knockin*^ mice and controls (adjusted *P* < 0.05) (see Table S1 for genes shown in the heat map in Fig. 6b). Biological process (GO), KEGG and mammalian phenotype enrichment analysis of the DEGs revealed that *Gprc6a-*^*KGKY-knockin*^ in liver resulted in differences in lipid and glucose metabolism (Fig. 6c–e). Genes induced in *Gprc6a-*^*KGKY-knockin*^ mice included genes involved in lipid homeostasis (6 genes), localization (20 genes), modification (7 genes), transport (14 genes), storage (10 genes), and fatty metabolic process (13 genes) (Fig. 6c). The complete list of up and down regulated genes are shown in Tables S2 and S3 in Supplemental materials.

## Discussion

In the current studies, we used fine-scale genomic humanization of the GPRC6A mouse 3rd ICL to create a transgenic mouse with knock-in of the uniquely human KGKY polymorphism (*Gprc6a-*^*KGKY-knockin*^ mice) to determine if this polymorphism has a critical impact on the function of this G-protein coupled receptor in vivo. We found that *Gprc6a-*^*KGKY-knockin*^ mice differed from wild type mice expressing the ancestral RKLP variant by the presence of a lower circulating blood glucose, improved glucose tolerance test, increased serum insulin and FGF-21 concentrations. We also observed higher glucose levels in *Gprc6a-*^*KGKY-knockin*^ after either pyruvate or insulin administration. Thus, the GPRC6A*-*^KGKY^ polymorphism may enhance liver gluconeogenesis and glucose production as well as increase peripheral glucose utilization. We also observed a decrease white fat mass in *Gprc6a-*^*KGKY-knockin*^ mice, consistent with either direct or indirect effects of GPRC6A to regulate adipocyte function. Overall, these findings are consistent with the KGKY variant being a gain-of-function polymorphism.

Indeed, many of these phenotypic features mimic the effects of pharmacological activation of GPRC6A by Ocn treatment, which has been shown to stimulate insulin production by pancreatic β-cells, to enhance glucose and FGF-21 production by hepatocytes, to enhance glucose uptake by skeletal muscle without affecting insulin sensitivity, and to attenuate high fat diet induced hepatosteatosis in mice^21–23,29, 34–36^. A gain-of-function is also consistent with the recent in vitro findings showing that both hGPRC6A^ICL3_KGKY^ and humanized mouse mGPRC6A^ICL3_KGKY^ are retained intracellularly in ligand naive cells, but exhibit enhanced signaling responses in response to ligand stimulation^13,32,36^.

GPRC6A is expressed in several tissues, including β-cells, skeletal muscle, adipocytes and liver^17,29^, where it has direct effects to regulate glucose and fat metabolism. In this regard, GPRC6A is shown to have function in β-cell^8,9,18,19^, skeletal muscle^19^, testes^21^, skin^11^, adipocytes^6,19^, and fibroblasts^37^. Expression of the *GPRC6A-*^*KGKY*^ variant in the *Gprc6a-*^*KGKY-knockin*^ mouse under control of the endogenous promoter would be expected to express the gain-of-function polymorphism in multiple tissues. Our data suggests that GPRC6A also has important functions in the liver. Though additional studies in hepatocytes are needed, the hepatic transcriptome analysis suggests that the *GPRC6A-*^*KGKY*^ variant may directly affect hepatic glucose metabolism by decreasing glycolysis and increasing gluconeogenesis. GPRC6A also likely regulates hepatic glycogen metabolism by increasing glycogenesis and glycogenolysis. Our findings also implicate GPRC6A in hepatic fat metabolism where the alterations in gene expression suggests an effect to decrease fatty acid uptake and fatty acid synthesis, and to increase β-oxidation. Overall, these changes on glucose and lipid metabolism in the liver are consistent with the effects of Ocn activation of GPRC6A in the liver to prevent high-fat diet induced hepatosteatosis^17,29,36,38^ and effects of Ocn to stimulate hepatic glucose production and skeletal muscle glucose uptake and utilization through GPRC6A-dependent mechanisms^22^.

Another mechanism whereby GPRC6A regulates systemic energy homeostasis is through the release of an ensemble of hormones, including insulin secretion in pancreatic β-cells^3,7,9,17–19^, testosterone (T) production in Leydig cells^2,4,21,32^, IL-6 secretion in skeletal muscle^20,22^, adiponectin^6^ and lipocalin 2 from adipocytes^39^ and glucagon-like peptide 1 (GLP-1) production from intestinal cells^7,40,41^. The increased circulating levels of insulin in *Gprc6a-*^*KGKY-knockin*^ are consistent with GPRC6A stimulation of insulin secretion. In addition, we show for the first time that the hormone FGF-21 is regulated by GPRC6A. We have the novel finding of elevated FGF-21 levels in *Gprc6a-*^*KGKY-knockin*^ mice. Moreover, treatment of wild type mice with the GPRC6A ligand Ocn resulted increased FGF-21. Since Ocn is purported to be the cognate ligand for GPRC6A^4–9,21^, these findings are consistent with direct hepatic effects of GPRC6A to regulate FGF-21 production. Some of the metabolic alterations in *Gprc6a-*^*KGKY-knockin*^ mice may be due to FGF-21. FGF-21 has paracrine effects in the liver to regulate hepatic lipid oxidation, triglyceride clearance, ketogenesis, and gluconeogenesis^42,43^, as well as systemic effects to increase fat browning, glucose and fatty acid utilization and insulin sensitivity in muscle, to increase insulin synthesis^44^, and central nervous system actions to regulated energy intake and sugar consumption^38^. Limitations of our studies include the lack of information on the effects of dietary fat and calorie intake on the observed phenotype in *Gprc6a-*^*KGKY-knockin*^ mice.

The current study also addresses the controversy surrounding the function of the ICL3_KGKY polymorphism in GPRC6A in humans, which has been reported to be a hypomorphic by some investigators^26,31^. Our findings showing a gain-of-function in GPRC6A*-*^KGKY^ mice are consistent with in vitro data showing that the endogenous human GPRC6A containing the KGKY variant in the 3rd intracellular loop is a gain-of-function polymorphism in PC-3 cells in vitro^13,32^. The GPRC6A*-*^KGKY^ variant as a risk factor for metabolic syndrome, however, has not been well studied in human populations, and case controlled genetic associative studies have been inadequately powered to reach a definitive conclusion^26^; although the P91S (rs2274911) SNP is associated with insulin resistance^30,45^. Clinical association studies, however, showing that levels of Ocn, are inversely associated with glycemic status and insulin^46^, body mass index, fasting glucose and insulin, triglycerides, and leptin, and positively correlated with adiponectin in humans^33,47^, suggest that GPRC6A is functional in humans. Additional studies are needed to understand the differential functions of GPRC6A*-*^KGKY^ GPRC6A*-*^RKLP^ variants in humans. If GPRC6A is an important regulator of glucose and fat metabolism in humans, the unequally distributed RKLP and KGKY polymorphisms may impact racial differences in energy metabolism and help to explain the large variations in serum FGF-21 levels^48^.

In conclusion, the emerging direct functions of GPRC6A in the liver and other tissues, its ability to coordinate the release of an ensemble of metabolically active hormones, and the ability of multiple ligands, including amino acids, cations, Ocn, T and certain natural products to activate GPRC6A, suggests activation of this G-protein coupled receptor provides a new schema for understanding and manipulating energy metabolism. Collectively, the functions of GPRC6A may be to directly and indirectly coordinate the anabolic responses of liver, muscle, fat in response to diverse hormonal and environmental factors. If so, activation of GPRC6A may provide a target to treat metabolic syndrome (MetS), type 2 diabetes (T2D), and non-alcoholic fatty liver disease (NAFLD). To this end, small molecule agonists for GPRC6A have recently been developed that lower glucose in mice^49^ that may serve as therapeutic leads to develop GPRC6A agonists.

## Methods

### Animals

*Gprc6a-*^*KGKY-knockin*^ mice were generated by sgRNA/CRISPR-Cas9 target site in proximity to the RKLP → KY mutation site in The University of Alabama at Birmingham. A sgRNA targeting the site immediately preceding the RKLP region (GCTTTGTATTTGCATT-CAAG<GGG>) was selected for co-injection with Cas9 protein and a single stranded DNA oligonucleotide (ssODN-HDR; MmGprc6aHDRssODN: GGTAGTATAGACAGGGATGAATGTGATCCAAGCTATGAAGTAAATCAGCATCCCAAAGGTCAGAACTTGGCTTCGTTGTAATTCTCgtaTTTCCCtTTaAATGCAAATACAAAGCAAATGAAGGCCAGAACTGTGATGTAGCCCAGCATGGTACCAAATGCCAGTGCTGACCCCTCCTCACATTCCAGGAT) repair template. The ssODN was modified to replace the mouse-specific RKLP coding sequence to the human-specific KGKY coding sequence in ICL3, and also to ensure that the modified sequence is not targeted by the CRISPR/sgRNA. PCR genotyping using MmGprc6a-gen-F2 and MmGprc6a-gen-R1 primer set. The knockin of the human *GPRC6A* KY polymorphism PCR product cut by Dra I resulted in 335 and 242 bp bands.

Mice were maintained and used in accordance with recommendations as described (National Research Council. 1985; Guide for the Care and Use of Laboratory Animals DHHS Publication NIH 86-23, Institute on Laboratory Animal Resources, Rockville, MD, USA) and following guidelines established by the University of Tennessee Health Science Center Institutional Animal Care and Use Committee. The animal study protocol was approved by the institutional review boards at University of Tennessee Health Science Center Institutional Animal Care and Use Committee.

### Site-directed mutagenesis

Site-directed mutagenesis was conducted using the QuickChange mutagenesis kit (Stratagene) according to the protocols of the manufacturer using a primer set including mGPRC6A.KYfor: gcattcaagggcaaatatgagaattacaacgaagcc and mGPRC6A.KYrev: ggcttcgttgtaattctcatatttgcccttgaatgc. Mutant was constructed on wild type mouse GPRC6A (mGPRC6A^ICL3_KGRKLP^ in pcDNA3 vector) background. The mGPRC6A^ICL3_KGKY^ was confirmed by DNA sequencing. HEK-293 cells were transfected with mGPRC6A^ICL3_KGRKLP^ or mGPRC6A^ICL3_KGKY^ plasmids using TransFast Transfection Reagent (Promega) for 48 h, then the transfected cells were selected by G418.

### Measurement of total and phospho‐ERK and -mTOR by Elisa analysis

Briefly, HEK‐293 cells transfected with wild type mouse GPRC6A (mGPRC6A^ICL3_KGRKLP^) and mGPRC6A^ICL3_KGKY^ mutant cDNA plasmids were starved by overnight incubation in serum‐free DMEM/F12 containing 0.1% bovine serum albumin (BSA) and stimulated with various ligands at different doses. ERK activation were assessed the time as indicated after treatment by using ERK1/2 (phospho‐T203/Y204) ELISA Kit (Invitrogen) corrected for the amount of total ERK using ERK1/2 (Total) ELISA Kit (Invitrogen) to measure ERK levels. mTOR activation were assessed by using mTOR ELISA Kit (abcam).

### Metabolic studies

The glucose tolerance test (GTT) was performed by injecting glucose (2 g/kg body weight) intraperitoneally^50^ after a 5 h fast, and monitoring blood glucose using glucose strips and the Accu-Check glucometer at the indicated times^51^. For the insulin tolerance test^33^, mice were fasted for 5 h, injected IP with insulin (0.75 U/kg body weight, Sigma; St. Louis, MO, USA), and blood glucose levels were measured at indicated times as described^24^. For the pyruvate tolerance test (PTT), samples were collected following IP injection with pyruvate sodium (2 g/kg bodyweight) to 5 h fasted mice. Insulin (mouse) ultrasensitive ELISA kit was obtained from ALPCO Immunoassays (Salem, NH, USA). Glycogen assay and cholesterol quantitation kits were purchased from Sigma (St. Louis, MO, USA). Triglyceride colorimetric assay kit was obtained from Cayman chemical (Ann Arbor, MI, USA). Free fatty acid assay kit was purchased from Fisher Scientific (Pittsburgh, PA, USA). Rat/Mouse FGF-21 Elisa kit purchased from EMD Millipore (Burlington, MA, USA).

### RNA extraction

Total RNA was extracted from mouse liver tissue (~ 30 mg for each sample) using QIAGEN RNeasy Mini Kit (Frederick, MD, USA). QIAGEN RNase-free DNase Set (Frederick, MD, USA) were used for RNA cleanup. RNA quantity was determined by Qubit fluorometer and RNA integrity were determined by Agilent 2100 bioanalyzer (Santa Clara, CA, USA). The qualified samples (RNA integrity number, RIN > 9) were subjected to RNA-seq analysis.

### Real-time RT-PCR

We used 2.0 μg of total RNAs for reverse transcription using cDNA synthesis kit (Bio-Rad). PCR reactions were described in previously publications^9,17^. The primers for mouse *Fgf21* (NM_020013) consisted of mFGF21-F67: CTGCTGGGGGTCTACCAAG and mFGF21-R220: CTGCGCCTACCACTGTTCC, and for the *Cyclophilin A* (NM_008907) consisted of CycA.For: CTGCACTGCCAAGACTGAAT and CycA.Rev: CCACAATGTTCAT-GCCTTCT.

### Library preparation and sequencing

The library preparation and sequencing were carried out by Novogene Co., Ltd. (Chula Vista, CA, USA). Briefly, mRNA was first enriched using oligo^52^ beads and fragmented randomly by adding fragmentation buffer. Then the cDNA was synthesized by using mRNA template and random hexamers primer, after which a custom second-strand synthesis buffer (Illumina; Mountain View, CA, USA), dNTPs, RNase H, and DNA polymerase I were added to initiate the second-strand synthesis. Second, after terminal repair, a ligation and sequencing adaptor ligation, the double-stranded cDNA library was completed through size selection and PCR enrichment. The library quality was accessed by Qubit 2.0, Agilent 2100, and Q-PCR. The DNA from the qualified libraries are fed into Illumina sequencers at an average depth of 42 million reads per sample.

### RNA-seq data analysis

Raw reads were quality filtered with NGS QC Toolkit version 2.3^53^ to remove adaptor contaminated reads or reads containing > 20% low-quality (*Q* < 20) bases. Filtered reads were aligned to the mouse reference sequence (GRCm38/mm10) using STAR aligner version 2.5.0a^54^. Raw read count was quantified across all annotated mm10 transcript using FeatureCounts version 1.6.3 implemented in Subread package^55^, then submitted to DeSeq2 version 1.10.1^56^ to identify the differentially expressed genes between *Gprc6a-*^*KGKY-knockin*^ and WT groups (three replicates for each group). Differentially expressed genes were defined as having an adjusted P value < 0.05. Gene set enrichment analysis for Gene Ontology (GO), Kyoto Encyclopedia of Genes and Genomes (KEGG), and Mammalian Phenotype Ontology were analyzed with WebGestalt^57^ (https://www.webgestalt.org/) with default parameters. A threshold of FDR < 0.05 was used to determine the significant enriched terms. Volcano plot and heatmaps were drawn using R program.

### Statistics

We evaluated differences between groups by Student’s *t* test, and for multiple groups by two-way ANOVA, followed by a post-hoc Tukey's test. Significance was set at P < 0.05. All values are expressed as means ± SEM. All computations were performed using the Statgraphic statistical graphics system (STSC Inc., Rockville, MD, USA).

## Supplementary information

Supplementary information.
