# Supplementary material for: Humanized GPRC6AKGKY is a gain-of-function polymorphism in mice
Source: Sci Rep. 2020 Jul 7;10:11143. doi: 10.1038/s41598-020-68113-z (PMC7341878; doi:10.1038/s41598-020-68113-z)
Supplement: Supplementary file 1 — Supplementary information. [file 41598_2020_68113_MOESM1_ESM.docx]

**Supplemental information**

**Humanized *GPRC6A^KGKY^* is a gain-of-function polymorphism in mice**

Min Pi^1 *^, Fuyi Xu^2^, Ruisong Ye^1^, Satoru K. Nishimoto^3^, Robert A. Kesterson^4^, Robert W. Williams^2^, Lu Lu^2^, L. Darryl Quarles^1 *^

^1^Department of Medicine, ^2^Department of Genetics, Genomics and Informatics**,** and ^3^Department of Microbiology, Immunology and Biochemistry,

University of Tennessee Health Science Center, 19 S Manassas St. Memphis, TN 38163

^4^ Department of Genetics, University of Alabama at Birmingham, 720 20^th^ Street South, Birmingham, AL 35294

**
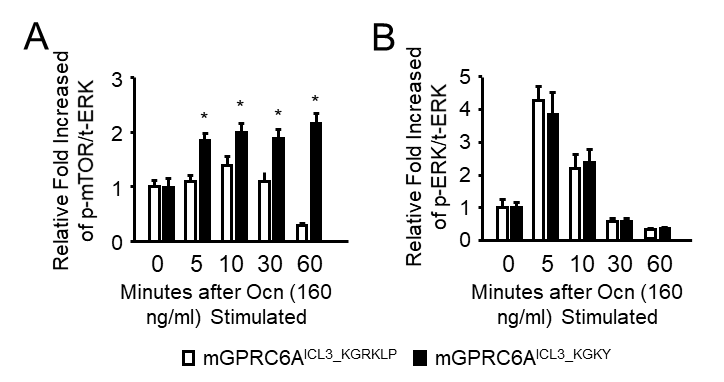
**

**S1 Figure. Humanized *GPRC6A^KGKY^* is a gain-of-function polymorphism**. The time course for mGPRC6A^ICL3_KGRKLP^ and mGPRC6A^ICL3_KGKY^ mutant stimulated by 160 ng/ml Ocn as assessed by phosphorylation of mTOR (A) and ERK (B). HEK-293 cells were stably transfected with wild type mouse GPRC6A (mGPRC6A^ICL3_KGRKLP^) and mGPRC6A^ICL3_KGKY^ mutant, and assessed at different times (as indicated) after addition of Ocn at concentration of 160 ng/ml. Data are presented as mean ± SEM. *, significant difference from wild type mouse GPRC6A (mGPRC6A^ICL3_KGRKLP^) and mGPRC6A^ICL3_KGKY^ mutant (*P* < 0.05, Student’s *t* test; n = 6).

**S1 Table. Gene list fo heat map in *Gprc6a^_KGKY-knockin^*** **mice.**

A total of 359 (197 upregulated and 162 downregulated) genes that expressed in livers from *Gprc6a^_KGKY-knockin^* mice and controls were listed in a heatmap (figure 6a).

| **Ensembl Gene ID** | **Gene name** | **Chromosome** | **Gene start (bp)** | **Gene end (bp)** | **ky vs WT-log2FoldChange** | **ky vs WT-pvalue** | **ky vs WT-padj** |
| --- | --- | --- | --- | --- | --- | --- | --- |
| ENSMUSG00000032080 | Apoa4 | 9 | 46240696 | 46243459 | -1.85 | 4.25E-16 | 4.25E-13 |
| ENSMUSG00000039519 | Cyp7b1 | 3 | 18071950 | 18243338 | -1.26 | 4.79E-08 | 1.20E-05 |
| ENSMUSG00000067279 | Ppp1r3c | 19 | 36731737 | 36736653 | -1.16 | 5.74E-10 | 2.13E-07 |
| ENSMUSG00000035561 | Aldh1b1 | 4 | 45799022 | 45804604 | -0.95 | 3.01E-09 | 9.64E-07 |
| ENSMUSG00000030703 | Gdpd3 | 7 | 126766334 | 126775649 | -8.57 | 5.29E-29 | 9.40E-26 |
| ENSMUSG00000025003 | Cyp2c39 | 19 | 39510862 | 39568529 | -4.69 | 4.80E-09 | 1.39E-06 |
| ENSMUSG00000001642 | Akr1b3 | 6 | 34302434 | 34317478 | -1.92 | 3.94E-04 | 2.25E-02 |
| ENSMUSG00000049313 | Sorl1 | 9 | 41964720 | 42124297 | -1.82 | 2.36E-10 | 8.98E-08 |
| ENSMUSG00000031565 | Fgfr1 | 8 | 25513654 | 25575718 | -1.49 | 1.20E-11 | 5.82E-09 |
| ENSMUSG00000031441 | Atp11a | 8 | 12757014 | 12868728 | -1.34 | 1.32E-07 | 2.79E-05 |
| ENSMUSG00000060613 | Cyp2c70 | 19 | 40153353 | 40187333 | -1.19 | 6.43E-19 | 8.57E-16 |
| ENSMUSG00000020623 | Map2k6 | 11 | 110399122 | 110525522 | -0.87 | 7.80E-04 | 3.84E-02 |
| ENSMUSG00000025044 | Msr1 | 8 | 39581685 | 39642673 | -0.87 | 1.36E-04 | 9.76E-03 |
| ENSMUSG00000068246 | Apol9b | 15 | 77729039 | 77736382 | -0.79 | 4.46E-04 | 2.46E-02 |
| ENSMUSG00000028195 | Cyr61 | 3 | 145646976 | 145649981 | -0.76 | 3.77E-06 | 5.20E-04 |
| ENSMUSG00000003500 | Impdh1 | 6 | 29200434 | 29216364 | -0.74 | 2.95E-04 | 1.76E-02 |
| ENSMUSG00000025350 | Rdh5 | 10 | 128913593 | 128922888 | -0.74 | 8.08E-05 | 6.21E-03 |
| ENSMUSG00000032263 | Bckdhb | 9 | 83925145 | 84124240 | -0.72 | 3.57E-11 | 1.58E-08 |
| ENSMUSG00000067225 | Cyp2c54 | 19 | 40037941 | 40073811 | -0.68 | 4.99E-04 | 2.66E-02 |
| ENSMUSG00000018800 | Abca5 | 11 | 110269369 | 110337716 | -0.66 | 9.52E-04 | 4.48E-02 |
| ENSMUSG00000025190 | Got1 | 19 | 43499752 | 43524605 | -0.59 | 1.42E-04 | 1.01E-02 |
| ENSMUSG00000027983 | Cyp2u1 | 3 | 131288441 | 131303227 | -0.55 | 9.77E-04 | 4.53E-02 |
| ENSMUSG00000042102 | Dmgdh | 13 | 93674433 | 93752833 | -0.49 | 6.66E-05 | 5.41E-03 |
| ENSMUSG00000040136 | Abcc8 | 7 | 46104523 | 46180033 | -5.53 | 2.89E-04 | 1.74E-02 |
| ENSMUSG00000027199 | Gatm | 2 | 122594467 | 122611303 | -0.77 | 4.26E-04 | 2.41E-02 |
| ENSMUSG00000074768 | Bhmt | 13 | 93616675 | 93637961 | -0.66 | 9.75E-05 | 7.43E-03 |
| ENSMUSG00000029752 | Asns | 6 | 7675169 | 7693254 | -3.26 | 1.74E-06 | 2.56E-04 |
| ENSMUSG00000043300 | B3galnt1 | 3 | 69574158 | 69598960 | -1.85 | 7.00E-04 | 3.50E-02 |
| ENSMUSG00000039347 | Atp6v0e2 | 6 | 48537615 | 48541801 | -1.15 | 6.71E-04 | 3.42E-02 |
| ENSMUSG00000061906 | Ugt2b38 | 5 | 87409942 | 87424203 | -0.85 | 4.99E-05 | 4.27E-03 |
| ENSMUSG00000022304 | Dpys | 15 | 39768487 | 39857470 | -0.62 | 1.57E-07 | 3.22E-05 |
| ENSMUSG00000022615 | Tymp | 15 | 89371931 | 89377039 | -0.62 | 2.91E-05 | 2.83E-03 |
| ENSMUSG00000057228 | Aadat | 8 | 60505932 | 60545677 | -0.62 | 3.29E-04 | 1.95E-02 |
| ENSMUSG00000026272 | Agxt | 1 | 93135240 | 93145421 | -0.39 | 6.80E-04 | 3.43E-02 |
| ENSMUSG00000023087 | Noct | 3 | 51224447 | 51251644 | -3.22 | 2.28E-78 | 3.65E-74 |
| ENSMUSG00000021508 | Cxcl14 | 13 | 56288643 | 56296551 | -1.41 | 1.99E-04 | 1.28E-02 |
| ENSMUSG00000026442 | Nfasc | 1 | 132564690 | 132741797 | -1.06 | 4.37E-04 | 2.44E-02 |
| ENSMUSG00000038147 | Cd84 | 1 | 171839697 | 171890718 | -1.05 | 2.06E-04 | 1.32E-02 |
| ENSMUSG00000020427 | Igfbp3 | 11 | 7206086 | 7213923 | -0.83 | 1.19E-04 | 8.84E-03 |
| ENSMUSG00000009772 | Nuak2 | 1 | 132316126 | 132333488 | -0.74 | 5.21E-05 | 4.37E-03 |
| ENSMUSG00000031431 | Tsc22d3 | X | 140539528 | 140600659 | -0.69 | 8.64E-04 | 4.19E-02 |
| ENSMUSG00000030650 | Tmc5 | 7 | 118597297 | 118675086 | -10.50 | 3.35E-15 | 2.82E-12 |
| ENSMUSG00000072476 | Gm9008 | 6 | 76495432 | 76497784 | -8.72 | 1.57E-12 | 8.67E-10 |
| ENSMUSG00000085666 | Gm9855 | 10 | 42054113 | 42055306 | -7.98 | 1.80E-13 | 1.20E-10 |
| ENSMUSG00000082329 | Gm14287 | 2 | 157528869 | 157528985 | -6.85 | 4.25E-07 | 7.55E-05 |
| ENSMUSG00000044976 | Wdr72 | 9 | 74110356 | 74283308 | -6.51 | 1.41E-07 | 2.92E-05 |
| ENSMUSG00000094497 | Gm8210 | 1 | 43189156 | 43189638 | -6.39 | 3.13E-19 | 4.55E-16 |
| ENSMUSG00000059751 | Rps3a3 | 13 | 108670603 | 108671397 | -5.96 | 2.20E-30 | 4.40E-27 |
| ENSMUSG00000023964 | Calcr | 6 | 3685680 | 3764714 | -5.39 | 1.25E-04 | 9.09E-03 |
| ENSMUSG00000056863 | Olfr702 | 7 | 106823489 | 106833893 | -4.98 | 4.78E-04 | 2.58E-02 |
| ENSMUSG00000058126 | Tpm3-rs7 | 14 | 113314608 | 113316754 | -4.66 | 5.19E-55 | 2.07E-51 |
| ENSMUSG00000079845 | Xlr4a | X | 73074345 | 73082478 | -4.50 | 2.14E-06 | 3.06E-04 |
| ENSMUSG00000082145 | Gm12312 | 11 | 70358138 | 70358781 | -4.50 | 1.77E-04 | 1.17E-02 |
| ENSMUSG00000059058 | Tma7-ps | 1 | 171711088 | 171711282 | -4.32 | 5.95E-47 | 1.90E-43 |
| ENSMUSG00000043773 | 1700048O20Rik | 9 | 121937223 | 121947016 | -4.23 | 6.66E-05 | 5.41E-03 |
| ENSMUSG00000010066 | Cacna2d2 | 9 | 107399612 | 107529343 | -4.10 | 1.03E-03 | 4.69E-02 |
| ENSMUSG00000086962 | Gm12248 | 11 | 58063552 | 58093134 | -3.87 | 1.33E-05 | 1.48E-03 |
| ENSMUSG00000107092 | Gm7993 | 5 | 95974240 | 95975623 | -3.84 | 9.49E-04 | 4.48E-02 |
| ENSMUSG00000097662 | Gm2093 | 15 | 3996039 | 4015858 | -3.70 | 1.64E-09 | 5.68E-07 |
| ENSMUSG00000080893 | Gm15920 | 5 | 130188395 | 130188796 | -3.48 | 8.11E-24 | 1.30E-20 |
| ENSMUSG00000103953 | Gm29718 | 1 | 136793034 | 136793510 | -3.35 | 6.29E-13 | 3.87E-10 |
| ENSMUSG00000076612 | Ighg2c | 12 | 113285325 | 113288932 | -3.32 | 2.08E-12 | 1.11E-09 |
| ENSMUSG00000028359 | Orm3 | 4 | 63356162 | 63359511 | -3.25 | 4.52E-11 | 1.90E-08 |
| ENSMUSG00000062611 | Rps3a2 | 14 | 88123017 | 88123808 | -3.24 | 2.58E-46 | 6.87E-43 |
| ENSMUSG00000050550 | Gm11868 | 4 | 18844202 | 18845679 | -3.17 | 1.39E-14 | 1.01E-11 |
| ENSMUSG00000063953 | Amd2 | 10 | 35708675 | 35711892 | -3.11 | 5.92E-18 | 6.77E-15 |
| ENSMUSG00000020185 | E2f7 | 10 | 110745439 | 110787384 | -3.09 | 6.17E-06 | 7.82E-04 |
| ENSMUSG00000084349 | Rpl3-ps1 | X | 13202571 | 13203780 | -3.06 | 1.46E-63 | 7.78E-60 |
| ENSMUSG00000093916 | Gm379 | X | 108664004 | 108664891 | -2.89 | 8.88E-04 | 4.26E-02 |
| ENSMUSG00000074280 | Gm6166 | 9 | 57483963 | 57484371 | -2.82 | 1.93E-09 | 6.43E-07 |
| ENSMUSG00000036744 | Olfr701 | 7 | 106814129 | 106821472 | -2.81 | 8.90E-04 | 4.26E-02 |
| ENSMUSG00000091476 | Catspere2 | 1 | 177983423 | 178172704 | -2.75 | 4.24E-07 | 7.55E-05 |
| ENSMUSG00000067608 | Pcna-ps2 | 19 | 9283238 | 9284494 | -2.58 | 7.79E-11 | 3.11E-08 |
| ENSMUSG00000028008 | Asic5 | 3 | 81982290 | 82021233 | -2.58 | 1.03E-37 | 2.36E-34 |
| ENSMUSG00000023968 | Crip3 | 17 | 46428926 | 46431776 | -2.49 | 7.67E-04 | 3.79E-02 |
| ENSMUSG00000097451 | Rian | 12 | 109603940 | 109661716 | -2.40 | 3.23E-04 | 1.92E-02 |
| ENSMUSG00000000739 | Sult5a1 | 8 | 123140192 | 123158315 | -2.40 | 4.75E-05 | 4.08E-03 |
| ENSMUSG00000068877 | Selenbp2 | 3 | 94693556 | 94704413 | -2.27 | 8.79E-06 | 1.04E-03 |
| ENSMUSG00000061540 | Orm2 | 4 | 63362449 | 63365878 | -2.25 | 2.81E-04 | 1.71E-02 |
| ENSMUSG00000083833 | Gm13841 | 5 | 115801677 | 115802159 | -2.15 | 7.40E-09 | 2.11E-06 |
| ENSMUSG00000053702 | Nebl | 2 | 17343909 | 17731464 | -2.11 | 7.82E-08 | 1.81E-05 |
| ENSMUSG00000067144 | Slc22a7 | 17 | 46432183 | 46438457 | -2.08 | 4.89E-18 | 6.02E-15 |
| ENSMUSG00000067149 | Jchain | 5 | 88519809 | 88527891 | -2.06 | 2.03E-08 | 5.43E-06 |
| ENSMUSG00000095385 | D630033O11Rik | 9 | 43243599 | 43280076 | -2.05 | 1.30E-06 | 2.01E-04 |
| ENSMUSG00000018648 | Dusp14 | 11 | 84048041 | 84069261 | -2.04 | 5.46E-04 | 2.84E-02 |
| ENSMUSG00000066705 | Fxyd6 | 9 | 45370185 | 45396159 | -2.02 | 1.18E-11 | 5.82E-09 |
| ENSMUSG00000081223 | Gm12247 | 11 | 58097104 | 58097773 | -1.91 | 9.44E-07 | 1.51E-04 |
| ENSMUSG00000029380 | Cxcl1 | 5 | 90891241 | 90893115 | -1.81 | 1.69E-07 | 3.38E-05 |
| ENSMUSG00000040483 | Xaf1 | 11 | 72301629 | 72313733 | -1.77 | 1.70E-09 | 5.79E-07 |
| ENSMUSG00000043753 | Dmrta1 | 4 | 89679436 | 89694772 | -1.77 | 4.35E-05 | 3.81E-03 |
| ENSMUSG00000024697 | Gna14 | 19 | 16435667 | 16610818 | -1.70 | 2.07E-08 | 5.43E-06 |
| ENSMUSG00000072568 | Fam84b | 15 | 60818994 | 60853778 | -1.65 | 3.99E-08 | 1.03E-05 |
| ENSMUSG00000076609 | Igkc | 6 | 70726435 | 70726966 | -1.64 | 8.94E-04 | 4.27E-02 |
| ENSMUSG00000054074 | Skida1 | 2 | 18040676 | 18049051 | -1.61 | 4.60E-05 | 3.98E-03 |
| ENSMUSG00000094156 | Sult2a7 | 7 | 14465051 | 14494230 | -1.53 | 6.70E-07 | 1.13E-04 |
| ENSMUSG00000046380 | Jrk | 15 | 74702301 | 74709535 | -1.44 | 5.09E-04 | 2.68E-02 |
| ENSMUSG00000003469 | Phyhip | 14 | 70457476 | 70468832 | -1.41 | 1.01E-03 | 4.66E-02 |
| ENSMUSG00000038984 | Tspyl5 | 15 | 33683875 | 33687884 | -1.41 | 1.87E-05 | 1.94E-03 |
| ENSMUSG00000102375 | A930036K24Rik | 9 | 107680082 | 107681281 | -1.40 | 4.57E-05 | 3.97E-03 |
| ENSMUSG00000085006 | BC021767 | 3 | 94661830 | 94670696 | -1.39 | 8.72E-05 | 6.67E-03 |
| ENSMUSG00000081058 | Hist2h3c2 | 3 | 96238108 | 96239127 | -1.37 | 1.21E-04 | 8.86E-03 |
| ENSMUSG00000022415 | Syngr1 | 15 | 80091334 | 80119501 | -1.37 | 7.89E-04 | 3.87E-02 |
| ENSMUSG00000001761 | Smo | 6 | 29735503 | 29761365 | -1.31 | 3.46E-13 | 2.21E-10 |
| ENSMUSG00000023031 | Cela1 | 15 | 100674421 | 100687921 | -1.24 | 1.31E-07 | 2.78E-05 |
| ENSMUSG00000104445 | Rhbg | 3 | 88242874 | 88254709 | -1.24 | 1.12E-05 | 1.28E-03 |
| ENSMUSG00000068101 | Cenpm | 15 | 82233779 | 82244748 | -1.17 | 1.68E-04 | 1.13E-02 |
| ENSMUSG00000054855 | Rnd1 | 15 | 98663421 | 98677461 | -1.13 | 1.67E-07 | 3.37E-05 |
| ENSMUSG00000024427 | Spry4 | 18 | 38586268 | 38601268 | -1.10 | 2.83E-05 | 2.78E-03 |
| ENSMUSG00000000567 | Sox9 | 11 | 112782224 | 112787760 | -1.08 | 1.64E-04 | 1.11E-02 |
| ENSMUSG00000045087 | S1pr5 | 9 | 21242912 | 21248443 | -1.02 | 1.22E-05 | 1.38E-03 |
| ENSMUSG00000027175 | Tcp11l1 | 2 | 104657288 | 104712169 | -1.01 | 1.23E-07 | 2.66E-05 |
| ENSMUSG00000018008 | Cyth4 | 15 | 78597047 | 78622019 | -0.98 | 5.26E-04 | 2.75E-02 |
| ENSMUSG00000027556 | Car1 | 3 | 14766216 | 14808368 | -0.96 | 5.05E-05 | 4.30E-03 |
| ENSMUSG00000050663 | Trhde | 10 | 114398823 | 114802307 | -0.95 | 3.77E-06 | 5.20E-04 |
| ENSMUSG00000002396 | Ocel1 | 8 | 71371298 | 71379361 | -0.94 | 2.59E-04 | 1.60E-02 |
| ENSMUSG00000046688 | Tifa | 3 | 127789805 | 127832164 | -0.92 | 5.03E-06 | 6.76E-04 |
| ENSMUSG00000030587 | 2200002D01Rik | 7 | 29246561 | 29248466 | -0.90 | 7.10E-06 | 8.60E-04 |
| ENSMUSG00000026117 | Zap70 | 1 | 36761798 | 36782818 | -0.88 | 3.63E-05 | 3.30E-03 |
| ENSMUSG00000049907 | Rasl11b | 5 | 74195286 | 74199481 | -0.88 | 1.01E-03 | 4.66E-02 |
| ENSMUSG00000039621 | Prex1 | 2 | 166566342 | 166713832 | -0.88 | 2.43E-04 | 1.51E-02 |
| ENSMUSG00000025498 | Irf7 | 7 | 141262706 | 141266481 | -0.86 | 5.95E-05 | 4.90E-03 |
| ENSMUSG00000071669 | Snx29 | 16 | 11322908 | 11755472 | -0.86 | 5.92E-06 | 7.63E-04 |
| ENSMUSG00000041729 | Coro2b | 9 | 62419492 | 62537044 | -0.85 | 6.74E-04 | 3.42E-02 |
| ENSMUSG00000057068 | Fam47e | 5 | 92555069 | 92591279 | -0.84 | 1.12E-04 | 8.38E-03 |
| ENSMUSG00000025964 | Adam23 | 1 | 63445891 | 63596276 | -0.82 | 3.71E-05 | 3.35E-03 |
| ENSMUSG00000029534 | St7 | 6 | 17692933 | 17943025 | -0.82 | 2.89E-04 | 1.74E-02 |
| ENSMUSG00000015854 | Cd5l | 3 | 87357881 | 87371073 | -0.81 | 4.28E-04 | 2.41E-02 |
| ENSMUSG00000021376 | Tpmt | 13 | 47022482 | 47044737 | -0.80 | 5.69E-05 | 4.71E-03 |
| ENSMUSG00000025880 | Smad7 | 18 | 75367529 | 75395935 | -0.78 | 6.51E-04 | 3.34E-02 |
| ENSMUSG00000053716 | Dusp7 | 9 | 106368632 | 106375724 | -0.75 | 7.37E-05 | 5.81E-03 |
| ENSMUSG00000025200 | Cwf19l1 | 19 | 44108644 | 44135876 | -0.75 | 2.23E-07 | 4.30E-05 |
| ENSMUSG00000019960 | Dusp6 | 10 | 99263231 | 99267489 | -0.73 | 4.63E-04 | 2.53E-02 |
| ENSMUSG00000044206 | Vsig4 | X | 96247203 | 96293438 | -0.73 | 1.02E-03 | 4.68E-02 |
| ENSMUSG00000039196 | Orm1 | 4 | 63344560 | 63348163 | -0.73 | 1.73E-06 | 2.56E-04 |
| ENSMUSG00000039246 | Lyplal1 | 1 | 186087731 | 186117310 | -0.72 | 1.42E-05 | 1.55E-03 |
| ENSMUSG00000014542 | Clec4f | 6 | 83644542 | 83656187 | -0.70 | 7.60E-05 | 5.93E-03 |
| ENSMUSG00000037621 | Atoh8 | 6 | 72206177 | 72235577 | -0.70 | 7.13E-04 | 3.55E-02 |
| ENSMUSG00000045854 | Lyrm2 | 4 | 32800253 | 32801559 | -0.69 | 1.69E-04 | 1.13E-02 |
| ENSMUSG00000006522 | Itih3 | 14 | 30908572 | 30923760 | -0.69 | 1.30E-04 | 9.43E-03 |
| ENSMUSG00000030091 | Nup210 | 6 | 91013068 | 91116829 | -0.69 | 1.90E-04 | 1.24E-02 |
| ENSMUSG00000040658 | Dnph1 | 17 | 46496711 | 46499624 | -0.69 | 4.72E-04 | 2.57E-02 |
| ENSMUSG00000037826 | Ppm1k | 6 | 57506502 | 57535468 | -0.67 | 1.54E-05 | 1.64E-03 |
| ENSMUSG00000055652 | Klhl25 | 7 | 75848310 | 75874131 | -0.67 | 1.45E-04 | 1.02E-02 |
| ENSMUSG00000038005 | Hpf1 | 8 | 60890418 | 60908671 | -0.67 | 3.35E-04 | 1.98E-02 |
| ENSMUSG00000032575 | Manf | 9 | 106838312 | 106891979 | -0.66 | 4.85E-04 | 2.60E-02 |
| ENSMUSG00000020108 | Ddit4 | 10 | 59949669 | 59951834 | -0.66 | 8.58E-04 | 4.17E-02 |
| ENSMUSG00000036867 | Smad6 | 9 | 63953076 | 64022059 | -0.64 | 4.60E-04 | 2.53E-02 |
| ENSMUSG00000040699 | Limd2 | 11 | 106156256 | 106160860 | -0.63 | 1.53E-05 | 1.64E-03 |
| ENSMUSG00000022149 | C9 | 15 | 6445327 | 6498751 | -0.60 | 1.49E-04 | 1.04E-02 |
| ENSMUSG00000034947 | Tmem106a | 11 | 101582242 | 101591788 | -0.58 | 9.66E-04 | 4.51E-02 |
| ENSMUSG00000022498 | Txndc11 | 16 | 11074911 | 11134650 | -0.56 | 1.63E-04 | 1.11E-02 |
| ENSMUSG00000051319 | 1500011K16Rik | 2 | 127791388 | 127792488 | -0.55 | 1.11E-03 | 4.96E-02 |
| ENSMUSG00000040413 | Timd2 | 11 | 46668960 | 46707061 | -0.55 | 8.95E-07 | 1.45E-04 |
| ENSMUSG00000055312 | Them7 | 2 | 105224320 | 105379796 | -0.54 | 4.25E-04 | 2.41E-02 |
| ENSMUSG00000040033 | Stat2 | 10 | 128270559 | 128292849 | -0.54 | 1.75E-05 | 1.85E-03 |
| ENSMUSG00000029625 | Cpsf4 | 5 | 145167213 | 145182041 | -0.53 | 1.57E-04 | 1.09E-02 |
| ENSMUSG00000036083 | Slc17a3 | 13 | 23839434 | 23860716 | -0.52 | 1.20E-04 | 8.85E-03 |
| ENSMUSG00000030895 | Hpx | 7 | 105591613 | 105600137 | -0.51 | 1.03E-03 | 4.69E-02 |
| ENSMUSG00000003355 | Fkbp11 | 15 | 98724366 | 98728198 | -0.49 | 9.10E-04 | 4.32E-02 |
| ENSMUSG00000022337 | Emc2 | 15 | 43477229 | 43527763 | -0.44 | 3.76E-04 | 2.16E-02 |
| ENSMUSG00000019433 | Gipc1 | 8 | 83652677 | 83664694 | -0.43 | 5.09E-04 | 2.68E-02 |
| ENSMUSG00000051716 | Apon | 10 | 128254096 | 128255896 | -0.42 | 3.08E-05 | 2.91E-03 |
| ENSMUSG00000032540 | Abhd5 | 9 | 122351608 | 122381524 | 0.44 | 1.00E-03 | 4.63E-02 |
| ENSMUSG00000022707 | Gbe1 | 16 | 70313949 | 70569716 | 0.54 | 3.54E-05 | 3.27E-03 |
| ENSMUSG00000002289 | Angptl4 | 17 | 33773750 | 33781575 | 0.54 | 4.44E-04 | 2.46E-02 |
| ENSMUSG00000041798 | Gck | 11 | 5900820 | 5950081 | 0.58 | 2.30E-04 | 1.45E-02 |
| ENSMUSG00000023073 | Slc10a2 | 8 | 5083219 | 5105351 | 0.69 | 1.73E-04 | 1.15E-02 |
| ENSMUSG00000035451 | Foxa1 | 12 | 57540628 | 57546916 | 0.71 | 2.27E-04 | 1.43E-02 |
| ENSMUSG00000024052 | Lpin2 | 17 | 71182560 | 71249817 | 0.77 | 5.86E-07 | 9.96E-05 |
| ENSMUSG00000042010 | Acacb | 5 | 114146535 | 114250761 | 0.82 | 7.91E-05 | 6.11E-03 |
| ENSMUSG00000024978 | Gpam | 19 | 55069734 | 55099451 | 0.89 | 1.22E-06 | 1.90E-04 |
| ENSMUSG00000029167 | Ppargc1a | 5 | 51454250 | 51567726 | 0.94 | 4.30E-04 | 2.42E-02 |
| ENSMUSG00000002944 | Cd36 | 5 | 17781690 | 17888801 | 0.99 | 1.56E-05 | 1.66E-03 |
| ENSMUSG00000000440 | Pparg | 6 | 115360951 | 115490399 | 1.21 | 3.99E-07 | 7.34E-05 |
| ENSMUSG00000007655 | Cav1 | 6 | 17306335 | 17341452 | 1.34 | 1.93E-04 | 1.26E-02 |
| ENSMUSG00000027035 | Cers6 | 2 | 68861441 | 69114282 | 1.40 | 3.58E-07 | 6.65E-05 |
| ENSMUSG00000028655 | Mfsd2a | 4 | 122946850 | 122961188 | 1.42 | 4.27E-04 | 2.41E-02 |
| ENSMUSG00000027533 | Fabp5 | 3 | 10012548 | 10016607 | 1.57 | 3.44E-05 | 3.20E-03 |
| ENSMUSG00000020593 | Lpin1 | 12 | 16535669 | 16610966 | 1.65 | 1.33E-06 | 2.01E-04 |
| ENSMUSG00000038418 | Egr1 | 18 | 34859823 | 34864984 | 2.59 | 2.80E-04 | 1.71E-02 |
| ENSMUSG00000024526 | Cidea | 18 | 67343564 | 67367794 | 4.25 | 6.29E-06 | 7.86E-04 |
| ENSMUSG00000058793 | Cds2 | 2 | 132263148 | 132312050 | 0.39 | 1.31E-04 | 9.44E-03 |
| ENSMUSG00000000594 | Gm2a | 11 | 55098115 | 55113029 | 0.43 | 9.64E-04 | 4.51E-02 |
| ENSMUSG00000063558 | Aox1 | 1 | 58029931 | 58106413 | 0.46 | 8.82E-04 | 4.25E-02 |
| ENSMUSG00000040997 | Abhd4 | 14 | 54254188 | 54270637 | 0.48 | 1.63E-04 | 1.11E-02 |
| ENSMUSG00000027761 | Aadac | 3 | 60025717 | 60040160 | 0.50 | 2.21E-05 | 2.21E-03 |
| ENSMUSG00000032802 | Srxn1 | 2 | 152105516 | 152111376 | 0.50 | 1.94E-04 | 1.26E-02 |
| ENSMUSG00000025037 | Maoa | X | 16619698 | 16687818 | 0.51 | 5.21E-04 | 2.73E-02 |
| ENSMUSG00000023019 | Gpd1 | 15 | 99717515 | 99725005 | 0.51 | 1.85E-04 | 1.22E-02 |
| ENSMUSG00000025232 | Hexa | 9 | 59539540 | 59565109 | 0.54 | 6.70E-04 | 3.42E-02 |
| ENSMUSG00000000168 | Dlat | 9 | 50634633 | 50659780 | 0.58 | 3.28E-05 | 3.07E-03 |
| ENSMUSG00000010936 | Vac14 | 8 | 110618585 | 110720398 | 0.59 | 7.36E-07 | 1.21E-04 |
| ENSMUSG00000051483 | Cbr1 | 16 | 93605853 | 93610505 | 0.59 | 7.10E-05 | 5.67E-03 |
| ENSMUSG00000056035 | Cyp3a11 | 5 | 145854426 | 145879964 | 0.60 | 1.46E-04 | 1.03E-02 |
| ENSMUSG00000020532 | Acaca | 11 | 84129672 | 84401651 | 0.62 | 7.72E-05 | 5.99E-03 |
| ENSMUSG00000005225 | Plekha8 | 6 | 54595111 | 54645839 | 0.62 | 3.47E-04 | 2.04E-02 |
| ENSMUSG00000026456 | Cyb5r1 | 1 | 134405559 | 134411740 | 0.64 | 2.20E-04 | 1.40E-02 |
| ENSMUSG00000022940 | Pigp | 16 | 94358763 | 94371842 | 0.67 | 6.83E-04 | 3.43E-02 |
| ENSMUSG00000069456 | Rdh16 | 10 | 127801152 | 127846565 | 0.69 | 1.12E-03 | 4.99E-02 |
| ENSMUSG00000039745 | Htatip2 | 7 | 49759115 | 49773975 | 0.73 | 1.08E-06 | 1.72E-04 |
| ENSMUSG00000026692 | Fmo4 | 1 | 162793188 | 162813972 | 0.73 | 1.05E-03 | 4.75E-02 |
| ENSMUSG00000023963 | Cyp39a1 | 17 | 43667425 | 43751431 | 0.74 | 1.79E-04 | 1.18E-02 |
| ENSMUSG00000052151 | Plpp2 | 10 | 79526430 | 79533796 | 0.79 | 1.19E-07 | 2.61E-05 |
| ENSMUSG00000023805 | Synj2 | 17 | 5941280 | 6044290 | 0.81 | 2.98E-05 | 2.86E-03 |
| ENSMUSG00000024525 | Impa2 | 18 | 67289253 | 67319146 | 0.83 | 1.47E-05 | 1.60E-03 |
| ENSMUSG00000026853 | Crat | 2 | 30400471 | 30415813 | 0.87 | 1.17E-07 | 2.59E-05 |
| ENSMUSG00000003555 | Cyp17a1 | 19 | 46667165 | 46672974 | 0.91 | 4.70E-04 | 2.57E-02 |
| ENSMUSG00000042429 | Adora1 | 1 | 134199223 | 134235431 | 0.96 | 2.95E-04 | 1.76E-02 |
| ENSMUSG00000028995 | Fam126a | 5 | 23915276 | 24030690 | 1.01 | 7.78E-06 | 9.35E-04 |
| ENSMUSG00000032418 | Me1 | 9 | 86581371 | 86695953 | 1.07 | 1.35E-12 | 7.99E-10 |
| ENSMUSG00000031400 | G6pdx | X | 74409483 | 74429194 | 1.13 | 2.70E-07 | 5.09E-05 |
| ENSMUSG00000021670 | Hmgcr | 13 | 96648967 | 96670936 | 1.15 | 9.12E-04 | 4.32E-02 |
| ENSMUSG00000056148 | Rdh9 | 10 | 127776386 | 127792697 | 1.25 | 1.71E-04 | 1.15E-02 |
| ENSMUSG00000029822 | Osbpl3 | 6 | 50293330 | 50456201 | 1.60 | 7.69E-07 | 1.25E-04 |
| ENSMUSG00000068220 | Lgals1 | 15 | 78926725 | 78930465 | 1.69 | 1.59E-13 | 1.10E-10 |
| ENSMUSG00000050423 | Ppp1r3g | 13 | 35958839 | 35970388 | 2.55 | 6.07E-05 | 4.98E-03 |
| ENSMUSG00000049721 | Gal3st1 | 11 | 3983636 | 3999326 | 2.59 | 5.96E-10 | 2.17E-07 |
| ENSMUSG00000008734 | Gprc5b | 7 | 118972047 | 118995211 | 4.26 | 1.48E-09 | 5.25E-07 |
| ENSMUSG00000002831 | Plin4 | 17 | 56100591 | 56109803 | 1.71 | 7.68E-11 | 3.11E-08 |
| ENSMUSG00000021957 | Tkt | 14 | 30548359 | 30574720 | 0.48 | 1.21E-05 | 1.37E-03 |
| ENSMUSG00000020444 | Guk1 | 11 | 59183875 | 59192212 | 0.48 | 1.07E-03 | 4.81E-02 |
| ENSMUSG00000026688 | Mgst3 | 1 | 167371966 | 167393841 | 0.66 | 7.20E-05 | 5.73E-03 |
| ENSMUSG00000058135 | Gstm1 | 3 | 108012255 | 108017973 | 0.70 | 7.11E-07 | 1.18E-04 |
| ENSMUSG00000035948 | Acss3 | 10 | 106933517 | 107123668 | 0.85 | 3.56E-04 | 2.08E-02 |
| ENSMUSG00000090175 | Ugt1a9 | 1 | 88070800 | 88218997 | 1.02 | 3.29E-05 | 3.07E-03 |
| ENSMUSG00000023044 | Csad | 15 | 102176999 | 102204724 | 1.41 | 2.44E-12 | 1.26E-09 |
| ENSMUSG00000025270 | Alas2 | X | 150547375 | 150570638 | 2.45 | 5.27E-75 | 4.21E-71 |
| ENSMUSG00000021236 | Entpd5 | 12 | 84373857 | 84409029 | 0.45 | 1.03E-04 | 7.76E-03 |
| ENSMUSG00000022620 | Arsa | 15 | 89472476 | 89477425 | 0.59 | 1.93E-05 | 1.99E-03 |
| ENSMUSG00000056501 | Cebpb | 2 | 167688915 | 167690418 | 0.69 | 5.68E-04 | 2.95E-02 |
| ENSMUSG00000030545 | Pex11a | 7 | 79735957 | 79743131 | 0.71 | 2.06E-08 | 5.43E-06 |
| ENSMUSG00000025612 | Bach1 | 16 | 87698945 | 87733346 | 0.75 | 1.33E-06 | 2.01E-04 |
| ENSMUSG00000056999 | Ide | 19 | 37268743 | 37337852 | 0.81 | 9.95E-08 | 2.24E-05 |
| ENSMUSG00000030762 | Aqp8 | 7 | 123462291 | 123468004 | 0.84 | 1.17E-04 | 8.72E-03 |
| ENSMUSG00000021838 | Samd4 | 14 | 46882854 | 47105815 | 0.89 | 7.38E-04 | 3.67E-02 |
| ENSMUSG00000042377 | Fam83g | 11 | 61684091 | 61709951 | 0.95 | 4.37E-06 | 5.92E-04 |
| ENSMUSG00000004098 | Col5a3 | 9 | 20770050 | 20815067 | 1.20 | 1.70E-06 | 2.55E-04 |
| ENSMUSG00000020429 | Igfbp1 | 11 | 7197782 | 7202546 | 1.85 | 8.81E-06 | 1.04E-03 |
| ENSMUSG00000011034 | Slc5a1 | 5 | 33104219 | 33162870 | 2.09 | 6.30E-04 | 3.24E-02 |
| ENSMUSG00000030278 | Cidec | 6 | 113424634 | 113435760 | 2.95 | 2.95E-05 | 2.86E-03 |
| ENSMUSG00000029368 | Alb | 5 | 90460897 | 90476602 | 0.37 | 3.91E-04 | 2.24E-02 |
| ENSMUSG00000021930 | Spryd7 | 14 | 61531993 | 61556886 | 0.46 | 1.95E-04 | 1.26E-02 |
| ENSMUSG00000058600 | Rpl30 | 15 | 34440505 | 34443640 | 0.47 | 5.17E-06 | 6.89E-04 |
| ENSMUSG00000036528 | Ppfibp2 | 7 | 107595207 | 107748583 | 0.48 | 2.39E-04 | 1.49E-02 |
| ENSMUSG00000055322 | Tns1 | 1 | 73910231 | 74124449 | 0.48 | 1.07E-04 | 8.05E-03 |
| ENSMUSG00000050390 | C77080 | 4 | 129219578 | 129261404 | 0.49 | 2.35E-04 | 1.47E-02 |
| ENSMUSG00000021285 | Ppp1r13b | 12 | 111828457 | 111908110 | 0.49 | 5.09E-04 | 2.68E-02 |
| ENSMUSG00000032112 | Trappc4 | 9 | 44403698 | 44407600 | 0.50 | 8.40E-04 | 4.11E-02 |
| ENSMUSG00000041957 | Pkp2 | 16 | 16213318 | 16272712 | 0.51 | 2.80E-04 | 1.71E-02 |
| ENSMUSG00000038058 | Nod1 | 6 | 54923949 | 54972612 | 0.51 | 8.99E-04 | 4.28E-02 |
| ENSMUSG00000049940 | Pgrmc2 | 3 | 41066326 | 41083046 | 0.53 | 7.44E-05 | 5.83E-03 |
| ENSMUSG00000044037 | Als2cl | 9 | 110879870 | 110900530 | 0.55 | 1.11E-03 | 4.96E-02 |
| ENSMUSG00000032679 | Cd59a | 2 | 104095801 | 104115354 | 0.56 | 2.98E-05 | 2.86E-03 |
| ENSMUSG00000090137 | Uba52 | 8 | 70508263 | 70510801 | 0.56 | 7.00E-04 | 3.50E-02 |
| ENSMUSG00000068566 | Myadm | 7 | 3289080 | 3300442 | 0.56 | 7.60E-04 | 3.76E-02 |
| ENSMUSG00000057982 | Zfp809 | 9 | 22225714 | 22243354 | 0.58 | 9.56E-04 | 4.48E-02 |
| ENSMUSG00000053646 | Plxnb1 | 9 | 109095389 | 109119917 | 0.59 | 7.00E-06 | 8.55E-04 |
| ENSMUSG00000019437 | Tlcd1 | 11 | 78176711 | 78181909 | 0.59 | 1.03E-03 | 4.69E-02 |
| ENSMUSG00000026478 | Lamc1 | 1 | 153218922 | 153332786 | 0.59 | 2.71E-04 | 1.67E-02 |
| ENSMUSG00000060429 | Sntb1 | 15 | 55636388 | 55906949 | 0.59 | 4.75E-04 | 2.57E-02 |
| ENSMUSG00000035441 | Myo1d | 11 | 80482126 | 80780025 | 0.61 | 9.19E-06 | 1.07E-03 |
| ENSMUSG00000038859 | Baiap2l1 | 5 | 144264526 | 144358112 | 0.61 | 2.12E-05 | 2.13E-03 |
| ENSMUSG00000035873 | Pawr | 10 | 108332121 | 108414240 | 0.61 | 7.37E-05 | 5.81E-03 |
| ENSMUSG00000032028 | Nxpe2 | 9 | 48318006 | 48353454 | 0.62 | 4.41E-04 | 2.45E-02 |
| ENSMUSG00000034361 | Cpne2 | 8 | 94532990 | 94570531 | 0.62 | 5.89E-04 | 3.05E-02 |
| ENSMUSG00000025792 | Slc25a10 | 11 | 120491840 | 120499187 | 0.63 | 3.62E-05 | 3.30E-03 |
| ENSMUSG00000046324 | Ermp1 | 19 | 29608214 | 29648415 | 0.63 | 8.21E-06 | 9.79E-04 |
| ENSMUSG00000035093 | Secisbp2l | 2 | 125736986 | 125782870 | 0.64 | 4.31E-05 | 3.78E-03 |
| ENSMUSG00000028081 | Rps3a1 | 3 | 86137940 | 86142702 | 0.65 | 3.38E-09 | 1.06E-06 |
| ENSMUSG00000005089 | Slc1a2 | 2 | 102658659 | 102790784 | 0.67 | 2.60E-05 | 2.59E-03 |
| ENSMUSG00000044026 | Slc35g1 | 19 | 38395980 | 38405607 | 0.67 | 9.98E-06 | 1.16E-03 |
| ENSMUSG00000029370 | Rassf6 | 5 | 90603076 | 90640657 | 0.68 | 1.40E-04 | 1.00E-02 |
| ENSMUSG00000032498 | Mlh1 | 9 | 111228228 | 111271791 | 0.69 | 1.80E-06 | 2.59E-04 |
| ENSMUSG00000001630 | Stk38l | 6 | 146724995 | 146778812 | 0.69 | 4.01E-06 | 5.49E-04 |
| ENSMUSG00000036854 | Hspb6 | 7 | 30552178 | 30555443 | 0.69 | 1.55E-04 | 1.08E-02 |
| ENSMUSG00000107369 | Gstm2-ps1 | 5 | 116632908 | 116633564 | 0.70 | 3.65E-04 | 2.12E-02 |
| ENSMUSG00000008090 | Fgfrl1 | 5 | 108692382 | 108706924 | 0.70 | 6.85E-05 | 5.53E-03 |
| ENSMUSG00000048758 | Rpl29 | 9 | 106429454 | 106431568 | 0.70 | 1.51E-11 | 7.10E-09 |
| ENSMUSG00000029189 | Sel1l3 | 5 | 53107083 | 53213927 | 0.74 | 1.39E-05 | 1.53E-03 |
| ENSMUSG00000024580 | Grpel2 | 18 | 61712440 | 61726331 | 0.74 | 1.79E-06 | 2.59E-04 |
| ENSMUSG00000086265 | Marcksl1-ps4 | 13 | 4248735 | 4249025 | 0.75 | 2.37E-04 | 1.48E-02 |
| ENSMUSG00000039809 | Gabbr2 | 4 | 46662305 | 46991873 | 0.75 | 5.38E-06 | 7.12E-04 |
| ENSMUSG00000049047 | Armcx3 | X | 134756595 | 134761455 | 0.75 | 3.70E-04 | 2.15E-02 |
| ENSMUSG00000003644 | Rps6ka1 | 4 | 133847290 | 133887797 | 0.77 | 6.40E-06 | 7.93E-04 |
| ENSMUSG00000019066 | Rab3d | 9 | 21907491 | 21918192 | 0.77 | 4.36E-07 | 7.66E-05 |
| ENSMUSG00000026074 | Map4k4 | 1 | 39900913 | 40026310 | 0.78 | 2.10E-04 | 1.34E-02 |
| ENSMUSG00000078350 | Smim1 | 4 | 154020470 | 154026230 | 0.78 | 1.67E-04 | 1.13E-02 |
| ENSMUSG00000024962 | Vegfb | 19 | 6982473 | 6987651 | 0.79 | 6.10E-06 | 7.80E-04 |
| ENSMUSG00000048537 | Phldb1 | 9 | 44686304 | 44735198 | 0.80 | 5.92E-06 | 7.63E-04 |
| ENSMUSG00000036661 | Dennd3 | 15 | 73512560 | 73572242 | 0.81 | 1.59E-04 | 1.10E-02 |
| ENSMUSG00000034837 | Gnat1 | 9 | 107674474 | 107679712 | 0.82 | 5.52E-06 | 7.24E-04 |
| ENSMUSG00000044197 | Gpr146 | 5 | 139377697 | 139396415 | 0.83 | 2.45E-07 | 4.67E-05 |
| ENSMUSG00000062545 | Tlr12 | 4 | 128615443 | 128618619 | 0.85 | 5.02E-04 | 2.67E-02 |
| ENSMUSG00000074170 | Plekhf1 | 7 | 38216972 | 38228016 | 0.86 | 2.11E-05 | 2.13E-03 |
| ENSMUSG00000037348 | Paqr7 | 4 | 134496697 | 134510235 | 0.87 | 4.13E-07 | 7.50E-05 |
| ENSMUSG00000006711 | D130043K22Rik | 13 | 24845135 | 24901270 | 0.88 | 5.75E-07 | 9.89E-05 |
| ENSMUSG00000035413 | Tmem98 | 11 | 80810175 | 80822033 | 0.89 | 5.37E-07 | 9.33E-05 |
| ENSMUSG00000049532 | Sall2 | 14 | 52311172 | 52328762 | 0.92 | 1.87E-05 | 1.94E-03 |
| ENSMUSG00000085882 | 2610507I01Rik | 11 | 59199836 | 59202385 | 0.92 | 8.77E-04 | 4.24E-02 |
| ENSMUSG00000053553 | 3110082I17Rik | 5 | 139359739 | 139460527 | 0.93 | 6.27E-06 | 7.86E-04 |
| ENSMUSG00000032482 | Cspg5 | 9 | 110243783 | 110262576 | 0.93 | 4.41E-04 | 2.45E-02 |
| ENSMUSG00000026890 | Lhx6 | 2 | 36081953 | 36105408 | 0.93 | 2.39E-06 | 3.38E-04 |
| ENSMUSG00000026979 | Psd4 | 2 | 24367580 | 24414954 | 0.93 | 3.80E-05 | 3.42E-03 |
| ENSMUSG00000074639 | Rdh16f2 | 10 | 127866474 | 127877317 | 0.93 | 4.04E-09 | 1.22E-06 |
| ENSMUSG00000024901 | Peli3 | 19 | 4930651 | 4943127 | 0.96 | 1.11E-03 | 4.96E-02 |
| ENSMUSG00000022723 | Crybg3 | 16 | 59490775 | 59600979 | 0.97 | 1.00E-04 | 7.60E-03 |
| ENSMUSG00000053886 | Sh2d4a | 8 | 68276567 | 68347699 | 0.99 | 1.59E-04 | 1.10E-02 |
| ENSMUSG00000022297 | Fzd6 | 15 | 39006034 | 39038188 | 1.02 | 2.76E-05 | 2.72E-03 |
| ENSMUSG00000050069 | Grem2 | 1 | 174833785 | 174921819 | 1.02 | 5.25E-05 | 4.37E-03 |
| ENSMUSG00000060548 | Tnfrsf19 | 14 | 60963875 | 61046490 | 1.05 | 4.80E-04 | 2.59E-02 |
| ENSMUSG00000052133 | Sema5b | 16 | 35541145 | 35664732 | 1.09 | 3.96E-05 | 3.52E-03 |
| ENSMUSG00000060227 | Casc4 | 2 | 121866970 | 121936220 | 1.15 | 2.82E-04 | 1.71E-02 |
| ENSMUSG00000032332 | Col12a1 | 9 | 79598991 | 79718831 | 1.16 | 1.07E-03 | 4.81E-02 |
| ENSMUSG00000020099 | Unc5b | 10 | 60762593 | 60831581 | 1.18 | 6.23E-08 | 1.49E-05 |
| ENSMUSG00000002059 | Rab34 | 11 | 78188430 | 78192193 | 1.19 | 1.24E-08 | 3.48E-06 |
| ENSMUSG00000002058 | Unc119 | 11 | 78343482 | 78349164 | 1.23 | 2.04E-07 | 4.03E-05 |
| ENSMUSG00000039470 | Zdhhc2 | 8 | 40423815 | 40510268 | 1.24 | 8.54E-04 | 4.16E-02 |
| ENSMUSG00000049353 | Rd3 | 1 | 191977370 | 191988283 | 1.25 | 6.00E-04 | 3.10E-02 |
| ENSMUSG00000029674 | Limk1 | 5 | 134656039 | 134688598 | 1.25 | 4.14E-05 | 3.66E-03 |
| ENSMUSG00000059810 | Rgs3 | 4 | 62559847 | 62704001 | 1.27 | 8.77E-08 | 2.00E-05 |
| ENSMUSG00000028476 | Reck | 4 | 43875530 | 43944806 | 1.27 | 1.97E-11 | 9.00E-09 |
| ENSMUSG00000008153 | Clstn3 | 6 | 124430759 | 124464794 | 1.27 | 4.40E-08 | 1.12E-05 |
| ENSMUSG00000021699 | Pde4d | 13 | 108449948 | 109953461 | 1.29 | 4.02E-09 | 1.22E-06 |
| ENSMUSG00000032577 | Mapkapk3 | 9 | 107254927 | 107289877 | 1.31 | 2.21E-07 | 4.30E-05 |
| ENSMUSG00000105987 | AI506816 | 5 | 23698296 | 23712667 | 1.36 | 5.07E-08 | 1.25E-05 |
| ENSMUSG00000020911 | Krt19 | 11 | 100140810 | 100148665 | 1.37 | 4.98E-04 | 2.66E-02 |
| ENSMUSG00000029869 | Ephb6 | 6 | 41605482 | 41620509 | 1.37 | 5.21E-05 | 4.37E-03 |
| ENSMUSG00000035914 | Cd276 | 9 | 58524298 | 58555437 | 1.41 | 1.38E-08 | 3.82E-06 |
| ENSMUSG00000028464 | Tpm2 | 4 | 43514711 | 43523765 | 1.43 | 5.72E-15 | 4.58E-12 |
| ENSMUSG00000027820 | Mme | 3 | 63241537 | 63386030 | 1.45 | 1.44E-12 | 8.20E-10 |
| ENSMUSG00000046160 | Olig1 | 16 | 91269772 | 91271933 | 1.46 | 4.52E-11 | 1.90E-08 |
| ENSMUSG00000069601 | Ank3 | 10 | 69398773 | 70027438 | 1.50 | 8.15E-11 | 3.18E-08 |
| ENSMUSG00000064247 | Plcxd1 | 5 | 110099969 | 110105953 | 1.51 | 1.27E-04 | 9.22E-03 |
| ENSMUSG00000070605 | Zfp992 | 4 | 146449023 | 146470292 | 1.52 | 1.27E-05 | 1.42E-03 |
| ENSMUSG00000097316 | Gm10516 | 1 | 192136896 | 192151026 | 1.54 | 9.73E-04 | 4.52E-02 |
| ENSMUSG00000024411 | Aqp4 | 18 | 15389394 | 15403684 | 1.54 | 3.95E-05 | 3.52E-03 |
| ENSMUSG00000086813 | Gm13657 | 2 | 75777188 | 75782306 | 1.59 | 1.50E-05 | 1.63E-03 |
| ENSMUSG00000030800 | Prss8 | 7 | 127925716 | 127930104 | 1.68 | 4.40E-09 | 1.30E-06 |
| ENSMUSG00000031861 | Lpar2 | 8 | 69822429 | 69831102 | 1.69 | 1.02E-05 | 1.17E-03 |
| ENSMUSG00000023908 | Pkmyt1 | 17 | 23726336 | 23736735 | 1.72 | 2.07E-05 | 2.11E-03 |
| ENSMUSG00000079494 | Nat8f5 | 6 | 85817218 | 85820972 | 1.72 | 3.00E-16 | 3.20E-13 |
| ENSMUSG00000032068 | Plet1 | 9 | 50494525 | 50505482 | 1.75 | 3.76E-04 | 2.16E-02 |
| ENSMUSG00000044071 | Fam19a2 | 10 | 123263996 | 123741204 | 1.77 | 6.78E-04 | 3.43E-02 |
| ENSMUSG00000028072 | Ntrk1 | 3 | 87778244 | 87795162 | 1.91 | 1.95E-05 | 2.00E-03 |
| ENSMUSG00000066153 | Mup21 | 4 | 62147932 | 62150863 | 1.92 | 8.47E-16 | 7.97E-13 |
| ENSMUSG00000028680 | Plk3 | 4 | 117128655 | 117133963 | 1.98 | 3.06E-05 | 2.91E-03 |
| ENSMUSG00000055675 | Kbtbd11 | 8 | 15011025 | 15033333 | 1.99 | 2.78E-09 | 9.06E-07 |
| ENSMUSG00000074213 | Gm10642 | 9 | 70656251 | 70657860 | 2.04 | 5.21E-05 | 4.37E-03 |
| ENSMUSG00000097815 | Gm26809 | 6 | 18844220 | 18847360 | 2.22 | 6.90E-05 | 5.54E-03 |
| ENSMUSG00000030137 | Tuba8 | 6 | 121210696 | 121226854 | 2.23 | 1.13E-06 | 1.77E-04 |
| ENSMUSG00000079495 | Nat8f6 | 6 | 85808024 | 85820954 | 2.24 | 6.57E-06 | 8.09E-04 |
| ENSMUSG00000044949 | Ubtd2 | 11 | 32455370 | 32516687 | 2.31 | 3.61E-04 | 2.11E-02 |
| ENSMUSG00000004631 | Sgce | 6 | 4674350 | 4747207 | 2.47 | 1.51E-15 | 1.34E-12 |
| ENSMUSG00000039878 | Slc39a5 | 10 | 128395931 | 128401229 | 2.82 | 6.10E-15 | 4.64E-12 |
| ENSMUSG00000042195 | Slc35f2 | 9 | 53771538 | 53818154 | 3.16 | 2.48E-06 | 3.48E-04 |
| ENSMUSG00000061780 | Cfd | 10 | 79890853 | 79892655 | 4.52 | 2.17E-04 | 1.38E-02 |
| ENSMUSG00000092035 | Peg10 | 6 | 4747306 | 4760517 | 5.58 | 7.52E-08 | 1.77E-05 |
| ENSMUSG00000054453 | Sytl5 | X | 9885622 | 9994543 | 5.74 | 5.70E-08 | 1.38E-05 |
| ENSMUSG00000049154 | Fam183b | 11 | 58792797 | 58801960 | 6.35 | 3.62E-05 | 3.30E-03 |

**S2 Table. Up-regulated gene list in *Gprc6a^_KGKY-knockin^*** **mice.**

The complete list of up-regulated genes in livers from *Gprc6a^_KGKY-knockin^* mice compared to controls.

| **Ensembl Gene ID** | **Gene name** | **Chromosome** | **Gene start (bp)** | **Gene end (bp)** | **ky vs WT-log2FoldChange** | **ky vs WT-pvalue** | **ky vs WT-padj** |
| --- | --- | --- | --- | --- | --- | --- | --- |
| ENSMUSG00000049154 | Fam183b | 11 | 58792797 | 58801960 | 6.35 | 3.62E-05 | 3.30E-03 |
| ENSMUSG00000054453 | Sytl5 | X | 9885622 | 9994543 | 5.74 | 5.70E-08 | 1.38E-05 |
| ENSMUSG00000092035 | Peg10 | 6 | 4747306 | 4760517 | 5.58 | 7.52E-08 | 1.77E-05 |
| ENSMUSG00000061780 | Cfd | 10 | 79890853 | 79892655 | 4.52 | 2.17E-04 | 1.38E-02 |
| ENSMUSG00000008734 | Gprc5b | 7 | 118972047 | 118995211 | 4.26 | 1.48E-09 | 5.25E-07 |
| ENSMUSG00000024526 | Cidea | 18 | 67343564 | 67367794 | 4.25 | 6.29E-06 | 7.86E-04 |
| ENSMUSG00000042195 | Slc35f2 | 9 | 53771538 | 53818154 | 3.16 | 2.48E-06 | 3.48E-04 |
| ENSMUSG00000030278 | Cidec | 6 | 113424634 | 113435760 | 2.95 | 2.95E-05 | 2.86E-03 |
| ENSMUSG00000039878 | Slc39a5 | 10 | 128395931 | 128401229 | 2.82 | 6.10E-15 | 4.64E-12 |
| ENSMUSG00000038418 | Egr1 | 18 | 34859823 | 34864984 | 2.59 | 2.80E-04 | 1.71E-02 |
| ENSMUSG00000049721 | Gal3st1 | 11 | 3983636 | 3999326 | 2.59 | 5.96E-10 | 2.17E-07 |
| ENSMUSG00000050423 | Ppp1r3g | 13 | 35958839 | 35970388 | 2.55 | 6.07E-05 | 4.98E-03 |
| ENSMUSG00000004631 | Sgce | 6 | 4674350 | 4747207 | 2.47 | 1.51E-15 | 1.34E-12 |
| ENSMUSG00000025270 | Alas2 | X | 150547375 | 150570638 | 2.45 | 5.27E-75 | 4.21E-71 |
| ENSMUSG00000044949 | Ubtd2 | 11 | 32455370 | 32516687 | 2.31 | 3.61E-04 | 2.11E-02 |
| ENSMUSG00000079495 | Nat8f6 | 6 | 85808024 | 85820954 | 2.24 | 6.57E-06 | 8.09E-04 |
| ENSMUSG00000030137 | Tuba8 | 6 | 121210696 | 121226854 | 2.23 | 1.13E-06 | 1.77E-04 |
| ENSMUSG00000097815 | Gm26809 | 6 | 18844220 | 18847360 | 2.22 | 6.90E-05 | 5.54E-03 |
| ENSMUSG00000011034 | Slc5a1 | 5 | 33104219 | 33162870 | 2.09 | 6.30E-04 | 3.24E-02 |
| ENSMUSG00000074213 | Gm10642 | 9 | 70656251 | 70657860 | 2.04 | 5.21E-05 | 4.37E-03 |
| ENSMUSG00000055675 | Kbtbd11 | 8 | 15011025 | 15033333 | 1.99 | 2.78E-09 | 9.06E-07 |
| ENSMUSG00000028680 | Plk3 | 4 | 117128655 | 117133963 | 1.98 | 3.06E-05 | 2.91E-03 |
| ENSMUSG00000066153 | Mup21 | 4 | 62147932 | 62150863 | 1.92 | 8.47E-16 | 7.97E-13 |
| ENSMUSG00000028072 | Ntrk1 | 3 | 87778244 | 87795162 | 1.91 | 1.95E-05 | 2.00E-03 |
| ENSMUSG00000020429 | Igfbp1 | 11 | 7197782 | 7202546 | 1.85 | 8.81E-06 | 1.04E-03 |
| ENSMUSG00000044071 | Fam19a2 | 10 | 123263996 | 123741204 | 1.77 | 6.78E-04 | 3.43E-02 |
| ENSMUSG00000032068 | Plet1 | 9 | 50494525 | 50505482 | 1.75 | 3.76E-04 | 2.16E-02 |
| ENSMUSG00000079494 | Nat8f5 | 6 | 85817218 | 85820972 | 1.72 | 3.00E-16 | 3.20E-13 |
| ENSMUSG00000023908 | Pkmyt1 | 17 | 23726336 | 23736735 | 1.72 | 2.07E-05 | 2.11E-03 |
| ENSMUSG00000002831 | Plin4 | 17 | 56100591 | 56109803 | 1.71 | 7.68E-11 | 3.11E-08 |
| ENSMUSG00000031861 | Lpar2 | 8 | 69822429 | 69831102 | 1.69 | 1.02E-05 | 1.17E-03 |
| ENSMUSG00000068220 | Lgals1 | 15 | 78926725 | 78930465 | 1.69 | 1.59E-13 | 1.10E-10 |
| ENSMUSG00000030800 | Prss8 | 7 | 127925716 | 127930104 | 1.68 | 4.40E-09 | 1.30E-06 |
| ENSMUSG00000020593 | Lpin1 | 12 | 16535669 | 16610966 | 1.65 | 1.33E-06 | 2.01E-04 |
| ENSMUSG00000029822 | Osbpl3 | 6 | 50293330 | 50456201 | 1.60 | 7.69E-07 | 1.25E-04 |
| ENSMUSG00000086813 | Gm13657 | 2 | 75777188 | 75782306 | 1.59 | 1.50E-05 | 1.63E-03 |
| ENSMUSG00000027533 | Fabp5 | 3 | 10012548 | 10016607 | 1.57 | 3.44E-05 | 3.20E-03 |
| ENSMUSG00000024411 | Aqp4 | 18 | 15389394 | 15403684 | 1.54 | 3.95E-05 | 3.52E-03 |
| ENSMUSG00000097316 | Gm10516 | 1 | 192136896 | 192151026 | 1.54 | 9.73E-04 | 4.52E-02 |
| ENSMUSG00000070605 | Zfp992 | 4 | 146449023 | 146470292 | 1.52 | 1.27E-05 | 1.42E-03 |
| ENSMUSG00000064247 | Plcxd1 | 5 | 110099969 | 110105953 | 1.51 | 1.27E-04 | 9.22E-03 |
| ENSMUSG00000069601 | Ank3 | 10 | 69398773 | 70027438 | 1.50 | 8.15E-11 | 3.18E-08 |
| ENSMUSG00000046160 | Olig1 | 16 | 91269772 | 91271933 | 1.46 | 4.52E-11 | 1.90E-08 |
| ENSMUSG00000027820 | Mme | 3 | 63241537 | 63386030 | 1.45 | 1.44E-12 | 8.20E-10 |
| ENSMUSG00000028464 | Tpm2 | 4 | 43514711 | 43523765 | 1.43 | 5.72E-15 | 4.58E-12 |
| ENSMUSG00000028655 | Mfsd2a | 4 | 122946850 | 122961188 | 1.42 | 4.27E-04 | 2.41E-02 |
| ENSMUSG00000023044 | Csad | 15 | 102176999 | 102204724 | 1.41 | 2.44E-12 | 1.26E-09 |
| ENSMUSG00000035914 | Cd276 | 9 | 58524298 | 58555437 | 1.41 | 1.38E-08 | 3.82E-06 |
| ENSMUSG00000027035 | Cers6 | 2 | 68861441 | 69114282 | 1.40 | 3.58E-07 | 6.65E-05 |
| ENSMUSG00000029869 | Ephb6 | 6 | 41605482 | 41620509 | 1.37 | 5.21E-05 | 4.37E-03 |
| ENSMUSG00000020911 | Krt19 | 11 | 100140810 | 100148665 | 1.37 | 4.98E-04 | 2.66E-02 |
| ENSMUSG00000105987 | AI506816 | 5 | 23698296 | 23712667 | 1.36 | 5.07E-08 | 1.25E-05 |
| ENSMUSG00000007655 | Cav1 | 6 | 17306335 | 17341452 | 1.34 | 1.93E-04 | 1.26E-02 |
| ENSMUSG00000032577 | Mapkapk3 | 9 | 107254927 | 107289877 | 1.31 | 2.21E-07 | 4.30E-05 |
| ENSMUSG00000021699 | Pde4d | 13 | 108449948 | 109953461 | 1.29 | 4.02E-09 | 1.22E-06 |
| ENSMUSG00000008153 | Clstn3 | 6 | 124430759 | 124464794 | 1.27 | 4.40E-08 | 1.12E-05 |
| ENSMUSG00000028476 | Reck | 4 | 43875530 | 43944806 | 1.27 | 1.97E-11 | 9.00E-09 |
| ENSMUSG00000059810 | Rgs3 | 4 | 62559847 | 62704001 | 1.27 | 8.77E-08 | 2.00E-05 |
| ENSMUSG00000029674 | Limk1 | 5 | 134656039 | 134688598 | 1.25 | 4.14E-05 | 3.66E-03 |
| ENSMUSG00000049353 | Rd3 | 1 | 191977370 | 191988283 | 1.25 | 6.00E-04 | 3.10E-02 |
| ENSMUSG00000056148 | Rdh9 | 10 | 127776386 | 127792697 | 1.25 | 1.71E-04 | 1.15E-02 |
| ENSMUSG00000039470 | Zdhhc2 | 8 | 40423815 | 40510268 | 1.24 | 8.54E-04 | 4.16E-02 |
| ENSMUSG00000002058 | Unc119 | 11 | 78343482 | 78349164 | 1.23 | 2.04E-07 | 4.03E-05 |
| ENSMUSG00000000440 | Pparg | 6 | 115360951 | 115490399 | 1.21 | 3.99E-07 | 7.34E-05 |
| ENSMUSG00000004098 | Col5a3 | 9 | 20770050 | 20815067 | 1.20 | 1.70E-06 | 2.55E-04 |
| ENSMUSG00000002059 | Rab34 | 11 | 78188430 | 78192193 | 1.19 | 1.24E-08 | 3.48E-06 |
| ENSMUSG00000020099 | Unc5b | 10 | 60762593 | 60831581 | 1.18 | 6.23E-08 | 1.49E-05 |
| ENSMUSG00000032332 | Col12a1 | 9 | 79598991 | 79718831 | 1.16 | 1.07E-03 | 4.81E-02 |
| ENSMUSG00000060227 | Casc4 | 2 | 121866970 | 121936220 | 1.15 | 2.82E-04 | 1.71E-02 |
| ENSMUSG00000021670 | Hmgcr | 13 | 96648967 | 96670936 | 1.15 | 9.12E-04 | 4.32E-02 |
| ENSMUSG00000031400 | G6pdx | X | 74409483 | 74429194 | 1.13 | 2.70E-07 | 5.09E-05 |
| ENSMUSG00000052133 | Sema5b | 16 | 35541145 | 35664732 | 1.09 | 3.96E-05 | 3.52E-03 |
| ENSMUSG00000032418 | Me1 | 9 | 86581371 | 86695953 | 1.07 | 1.35E-12 | 7.99E-10 |
| ENSMUSG00000060548 | Tnfrsf19 | 14 | 60963875 | 61046490 | 1.05 | 4.80E-04 | 2.59E-02 |
| ENSMUSG00000050069 | Grem2 | 1 | 174833785 | 174921819 | 1.02 | 5.25E-05 | 4.37E-03 |
| ENSMUSG00000090175 | Ugt1a9 | 1 | 88070800 | 88218997 | 1.02 | 3.29E-05 | 3.07E-03 |
| ENSMUSG00000022297 | Fzd6 | 15 | 39006034 | 39038188 | 1.02 | 2.76E-05 | 2.72E-03 |
| ENSMUSG00000028995 | Fam126a | 5 | 23915276 | 24030690 | 1.01 | 7.78E-06 | 9.35E-04 |
| ENSMUSG00000053886 | Sh2d4a | 8 | 68276567 | 68347699 | 0.99 | 1.59E-04 | 1.10E-02 |
| ENSMUSG00000002944 | Cd36 | 5 | 17781690 | 17888801 | 0.99 | 1.56E-05 | 1.66E-03 |
| ENSMUSG00000022723 | Crybg3 | 16 | 59490775 | 59600979 | 0.97 | 1.00E-04 | 7.60E-03 |
| ENSMUSG00000042429 | Adora1 | 1 | 134199223 | 134235431 | 0.96 | 2.95E-04 | 1.76E-02 |
| ENSMUSG00000024901 | Peli3 | 19 | 4930651 | 4943127 | 0.96 | 1.11E-03 | 4.96E-02 |
| ENSMUSG00000042377 | Fam83g | 11 | 61684091 | 61709951 | 0.95 | 4.37E-06 | 5.92E-04 |
| ENSMUSG00000029167 | Ppargc1a | 5 | 51454250 | 51567726 | 0.94 | 4.30E-04 | 2.42E-02 |
| ENSMUSG00000074639 | Rdh16f2 | 10 | 127866474 | 127877317 | 0.93 | 4.04E-09 | 1.22E-06 |
| ENSMUSG00000026979 | Psd4 | 2 | 24367580 | 24414954 | 0.93 | 3.80E-05 | 3.42E-03 |
| ENSMUSG00000026890 | Lhx6 | 2 | 36081953 | 36105408 | 0.93 | 2.39E-06 | 3.38E-04 |
| ENSMUSG00000032482 | Cspg5 | 9 | 110243783 | 110262576 | 0.93 | 4.41E-04 | 2.45E-02 |
| ENSMUSG00000053553 | 3110082I17Rik | 5 | 139359739 | 139460527 | 0.93 | 6.27E-06 | 7.86E-04 |
| ENSMUSG00000085882 | 2610507I01Rik | 11 | 59199836 | 59202385 | 0.92 | 8.77E-04 | 4.24E-02 |
| ENSMUSG00000049532 | Sall2 | 14 | 52311172 | 52328762 | 0.92 | 1.87E-05 | 1.94E-03 |
| ENSMUSG00000003555 | Cyp17a1 | 19 | 46667165 | 46672974 | 0.91 | 4.70E-04 | 2.57E-02 |
| ENSMUSG00000024978 | Gpam | 19 | 55069734 | 55099451 | 0.89 | 1.22E-06 | 1.90E-04 |
| ENSMUSG00000021838 | Samd4 | 14 | 46882854 | 47105815 | 0.89 | 7.38E-04 | 3.67E-02 |
| ENSMUSG00000035413 | Tmem98 | 11 | 80810175 | 80822033 | 0.89 | 5.37E-07 | 9.33E-05 |
| ENSMUSG00000006711 | D130043K22Rik | 13 | 24845135 | 24901270 | 0.88 | 5.75E-07 | 9.89E-05 |
| ENSMUSG00000026853 | Crat | 2 | 30400471 | 30415813 | 0.87 | 1.17E-07 | 2.59E-05 |
| ENSMUSG00000037348 | Paqr7 | 4 | 134496697 | 134510235 | 0.87 | 4.13E-07 | 7.50E-05 |
| ENSMUSG00000074170 | Plekhf1 | 7 | 38216972 | 38228016 | 0.86 | 2.11E-05 | 2.13E-03 |
| ENSMUSG00000035948 | Acss3 | 10 | 106933517 | 107123668 | 0.85 | 3.56E-04 | 2.08E-02 |
| ENSMUSG00000062545 | Tlr12 | 4 | 128615443 | 128618619 | 0.85 | 5.02E-04 | 2.67E-02 |
| ENSMUSG00000030762 | Aqp8 | 7 | 123462291 | 123468004 | 0.84 | 1.17E-04 | 8.72E-03 |
| ENSMUSG00000044197 | Gpr146 | 5 | 139377697 | 139396415 | 0.83 | 2.45E-07 | 4.67E-05 |
| ENSMUSG00000024525 | Impa2 | 18 | 67289253 | 67319146 | 0.83 | 1.47E-05 | 1.60E-03 |
| ENSMUSG00000042010 | Acacb | 5 | 114146535 | 114250761 | 0.82 | 7.91E-05 | 6.11E-03 |
| ENSMUSG00000034837 | Gnat1 | 9 | 107674474 | 107679712 | 0.82 | 5.52E-06 | 7.24E-04 |
| ENSMUSG00000056999 | Ide | 19 | 37268743 | 37337852 | 0.81 | 9.95E-08 | 2.24E-05 |
| ENSMUSG00000036661 | Dennd3 | 15 | 73512560 | 73572242 | 0.81 | 1.59E-04 | 1.10E-02 |
| ENSMUSG00000023805 | Synj2 | 17 | 5941280 | 6044290 | 0.81 | 2.98E-05 | 2.86E-03 |
| ENSMUSG00000048537 | Phldb1 | 9 | 44686304 | 44735198 | 0.80 | 5.92E-06 | 7.63E-04 |
| ENSMUSG00000052151 | Plpp2 | 10 | 79526430 | 79533796 | 0.79 | 1.19E-07 | 2.61E-05 |
| ENSMUSG00000024962 | Vegfb | 19 | 6982473 | 6987651 | 0.79 | 6.10E-06 | 7.80E-04 |
| ENSMUSG00000078350 | Smim1 | 4 | 154020470 | 154026230 | 0.78 | 1.67E-04 | 1.13E-02 |
| ENSMUSG00000026074 | Map4k4 | 1 | 39900913 | 40026310 | 0.78 | 2.10E-04 | 1.34E-02 |
| ENSMUSG00000024052 | Lpin2 | 17 | 71182560 | 71249817 | 0.77 | 5.86E-07 | 9.96E-05 |
| ENSMUSG00000019066 | Rab3d | 9 | 21907491 | 21918192 | 0.77 | 4.36E-07 | 7.66E-05 |
| ENSMUSG00000003644 | Rps6ka1 | 4 | 133847290 | 133887797 | 0.77 | 6.40E-06 | 7.93E-04 |
| ENSMUSG00000049047 | Armcx3 | X | 134756595 | 134761455 | 0.75 | 3.70E-04 | 2.15E-02 |
| ENSMUSG00000039809 | Gabbr2 | 4 | 46662305 | 46991873 | 0.75 | 5.38E-06 | 7.12E-04 |
| ENSMUSG00000025612 | Bach1 | 16 | 87698945 | 87733346 | 0.75 | 1.33E-06 | 2.01E-04 |
| ENSMUSG00000086265 | Marcksl1-ps4 | 13 | 4248735 | 4249025 | 0.75 | 2.37E-04 | 1.48E-02 |
| ENSMUSG00000023963 | Cyp39a1 | 17 | 43667425 | 43751431 | 0.74 | 1.79E-04 | 1.18E-02 |
| ENSMUSG00000024580 | Grpel2 | 18 | 61712440 | 61726331 | 0.74 | 1.79E-06 | 2.59E-04 |
| ENSMUSG00000029189 | Sel1l3 | 5 | 53107083 | 53213927 | 0.74 | 1.39E-05 | 1.53E-03 |
| ENSMUSG00000026692 | Fmo4 | 1 | 162793188 | 162813972 | 0.73 | 1.05E-03 | 4.75E-02 |
| ENSMUSG00000039745 | Htatip2 | 7 | 49759115 | 49773975 | 0.73 | 1.08E-06 | 1.72E-04 |
| ENSMUSG00000035451 | Foxa1 | 12 | 57540628 | 57546916 | 0.71 | 2.27E-04 | 1.43E-02 |
| ENSMUSG00000030545 | Pex11a | 7 | 79735957 | 79743131 | 0.71 | 2.06E-08 | 5.43E-06 |
| ENSMUSG00000058135 | Gstm1 | 3 | 108012255 | 108017973 | 0.70 | 7.11E-07 | 1.18E-04 |
| ENSMUSG00000048758 | Rpl29 | 9 | 106429454 | 106431568 | 0.70 | 1.51E-11 | 7.10E-09 |
| ENSMUSG00000008090 | Fgfrl1 | 5 | 108692382 | 108706924 | 0.70 | 6.85E-05 | 5.53E-03 |
| ENSMUSG00000107369 | Gstm2-ps1 | 5 | 116632908 | 116633564 | 0.70 | 3.65E-04 | 2.12E-02 |
| ENSMUSG00000056501 | Cebpb | 2 | 167688915 | 167690418 | 0.69 | 5.68E-04 | 2.95E-02 |
| ENSMUSG00000069456 | Rdh16 | 10 | 127801152 | 127846565 | 0.69 | 1.12E-03 | 4.99E-02 |
| ENSMUSG00000023073 | Slc10a2 | 8 | 5083219 | 5105351 | 0.69 | 1.73E-04 | 1.15E-02 |
| ENSMUSG00000036854 | Hspb6 | 7 | 30552178 | 30555443 | 0.69 | 1.55E-04 | 1.08E-02 |
| ENSMUSG00000001630 | Stk38l | 6 | 146724995 | 146778812 | 0.69 | 4.01E-06 | 5.49E-04 |
| ENSMUSG00000032498 | Mlh1 | 9 | 111228228 | 111271791 | 0.69 | 1.80E-06 | 2.59E-04 |
| ENSMUSG00000029370 | Rassf6 | 5 | 90603076 | 90640657 | 0.68 | 1.40E-04 | 1.00E-02 |
| ENSMUSG00000044026 | Slc35g1 | 19 | 38395980 | 38405607 | 0.67 | 9.98E-06 | 1.16E-03 |
| ENSMUSG00000022940 | Pigp | 16 | 94358763 | 94371842 | 0.67 | 6.83E-04 | 3.43E-02 |
| ENSMUSG00000005089 | Slc1a2 | 2 | 102658659 | 102790784 | 0.67 | 2.60E-05 | 2.59E-03 |
| ENSMUSG00000026688 | Mgst3 | 1 | 167371966 | 167393841 | 0.66 | 7.20E-05 | 5.73E-03 |
| ENSMUSG00000028081 | Rps3a1 | 3 | 86137940 | 86142702 | 0.65 | 3.38E-09 | 1.06E-06 |
| ENSMUSG00000035093 | Secisbp2l | 2 | 125736986 | 125782870 | 0.64 | 4.31E-05 | 3.78E-03 |
| ENSMUSG00000026456 | Cyb5r1 | 1 | 134405559 | 134411740 | 0.64 | 2.20E-04 | 1.40E-02 |
| ENSMUSG00000046324 | Ermp1 | 19 | 29608214 | 29648415 | 0.63 | 8.21E-06 | 9.79E-04 |
| ENSMUSG00000025792 | Slc25a10 | 11 | 120491840 | 120499187 | 0.63 | 3.62E-05 | 3.30E-03 |
| ENSMUSG00000034361 | Cpne2 | 8 | 94532990 | 94570531 | 0.62 | 5.89E-04 | 3.05E-02 |
| ENSMUSG00000032028 | Nxpe2 | 9 | 48318006 | 48353454 | 0.62 | 4.41E-04 | 2.45E-02 |
| ENSMUSG00000005225 | Plekha8 | 6 | 54595111 | 54645839 | 0.62 | 3.47E-04 | 2.04E-02 |
| ENSMUSG00000020532 | Acaca | 11 | 84129672 | 84401651 | 0.62 | 7.72E-05 | 5.99E-03 |
| ENSMUSG00000035873 | Pawr | 10 | 108332121 | 108414240 | 0.61 | 7.37E-05 | 5.81E-03 |
| ENSMUSG00000038859 | Baiap2l1 | 5 | 144264526 | 144358112 | 0.61 | 2.12E-05 | 2.13E-03 |
| ENSMUSG00000035441 | Myo1d | 11 | 80482126 | 80780025 | 0.61 | 9.19E-06 | 1.07E-03 |
| ENSMUSG00000056035 | Cyp3a11 | 5 | 145854426 | 145879964 | 0.60 | 1.46E-04 | 1.03E-02 |
| ENSMUSG00000051483 | Cbr1 | 16 | 93605853 | 93610505 | 0.59 | 7.10E-05 | 5.67E-03 |
| ENSMUSG00000022620 | Arsa | 15 | 89472476 | 89477425 | 0.59 | 1.93E-05 | 1.99E-03 |
| ENSMUSG00000060429 | Sntb1 | 15 | 55636388 | 55906949 | 0.59 | 4.75E-04 | 2.57E-02 |
| ENSMUSG00000026478 | Lamc1 | 1 | 153218922 | 153332786 | 0.59 | 2.71E-04 | 1.67E-02 |
| ENSMUSG00000019437 | Tlcd1 | 11 | 78176711 | 78181909 | 0.59 | 1.03E-03 | 4.69E-02 |
| ENSMUSG00000053646 | Plxnb1 | 9 | 109095389 | 109119917 | 0.59 | 7.00E-06 | 8.55E-04 |
| ENSMUSG00000010936 | Vac14 | 8 | 110618585 | 110720398 | 0.59 | 7.36E-07 | 1.21E-04 |
| ENSMUSG00000000168 | Dlat | 9 | 50634633 | 50659780 | 0.58 | 3.28E-05 | 3.07E-03 |
| ENSMUSG00000057982 | Zfp809 | 9 | 22225714 | 22243354 | 0.58 | 9.56E-04 | 4.48E-02 |
| ENSMUSG00000041798 | Gck | 11 | 5900820 | 5950081 | 0.58 | 2.30E-04 | 1.45E-02 |
| ENSMUSG00000068566 | Myadm | 7 | 3289080 | 3300442 | 0.56 | 7.60E-04 | 3.76E-02 |
| ENSMUSG00000090137 | Uba52 | 8 | 70508263 | 70510801 | 0.56 | 7.00E-04 | 3.50E-02 |
| ENSMUSG00000032679 | Cd59a | 2 | 104095801 | 104115354 | 0.56 | 2.98E-05 | 2.86E-03 |
| ENSMUSG00000044037 | Als2cl | 9 | 110879870 | 110900530 | 0.55 | 1.11E-03 | 4.96E-02 |
| ENSMUSG00000002289 | Angptl4 | 17 | 33773750 | 33781575 | 0.54 | 4.44E-04 | 2.46E-02 |
| ENSMUSG00000022707 | Gbe1 | 16 | 70313949 | 70569716 | 0.54 | 3.54E-05 | 3.27E-03 |
| ENSMUSG00000025232 | Hexa | 9 | 59539540 | 59565109 | 0.54 | 6.70E-04 | 3.42E-02 |
| ENSMUSG00000049940 | Pgrmc2 | 3 | 41066326 | 41083046 | 0.53 | 7.44E-05 | 5.83E-03 |
| ENSMUSG00000038058 | Nod1 | 6 | 54923949 | 54972612 | 0.51 | 8.99E-04 | 4.28E-02 |
| ENSMUSG00000023019 | Gpd1 | 15 | 99717515 | 99725005 | 0.51 | 1.85E-04 | 1.22E-02 |
| ENSMUSG00000025037 | Maoa | X | 16619698 | 16687818 | 0.51 | 5.21E-04 | 2.73E-02 |
| ENSMUSG00000041957 | Pkp2 | 16 | 16213318 | 16272712 | 0.51 | 2.80E-04 | 1.71E-02 |
| ENSMUSG00000032802 | Srxn1 | 2 | 152105516 | 152111376 | 0.50 | 1.94E-04 | 1.26E-02 |
| ENSMUSG00000027761 | Aadac | 3 | 60025717 | 60040160 | 0.50 | 2.21E-05 | 2.21E-03 |
| ENSMUSG00000032112 | Trappc4 | 9 | 44403698 | 44407600 | 0.50 | 8.40E-04 | 4.11E-02 |
| ENSMUSG00000021285 | Ppp1r13b | 12 | 111828457 | 111908110 | 0.49 | 5.09E-04 | 2.68E-02 |
| ENSMUSG00000050390 | C77080 | 4 | 129219578 | 129261404 | 0.49 | 2.35E-04 | 1.47E-02 |
| ENSMUSG00000020444 | Guk1 | 11 | 59183875 | 59192212 | 0.48 | 1.07E-03 | 4.81E-02 |
| ENSMUSG00000055322 | Tns1 | 1 | 73910231 | 74124449 | 0.48 | 1.07E-04 | 8.05E-03 |
| ENSMUSG00000036528 | Ppfibp2 | 7 | 107595207 | 107748583 | 0.48 | 2.39E-04 | 1.49E-02 |
| ENSMUSG00000040997 | Abhd4 | 14 | 54254188 | 54270637 | 0.48 | 1.63E-04 | 1.11E-02 |
| ENSMUSG00000021957 | Tkt | 14 | 30548359 | 30574720 | 0.48 | 1.21E-05 | 1.37E-03 |
| ENSMUSG00000058600 | Rpl30 | 15 | 34440505 | 34443640 | 0.47 | 5.17E-06 | 6.89E-04 |
| ENSMUSG00000063558 | Aox1 | 1 | 58029931 | 58106413 | 0.46 | 8.82E-04 | 4.25E-02 |
| ENSMUSG00000021930 | Spryd7 | 14 | 61531993 | 61556886 | 0.46 | 1.95E-04 | 1.26E-02 |
| ENSMUSG00000021236 | Entpd5 | 12 | 84373857 | 84409029 | 0.45 | 1.03E-04 | 7.76E-03 |
| ENSMUSG00000032540 | Abhd5 | 9 | 122351608 | 122381524 | 0.44 | 1.00E-03 | 4.63E-02 |
| ENSMUSG00000000594 | Gm2a | 11 | 55098115 | 55113029 | 0.43 | 9.64E-04 | 4.51E-02 |
| ENSMUSG00000058793 | Cds2 | 2 | 132263148 | 132312050 | 0.39 | 1.31E-04 | 9.44E-03 |
| ENSMUSG00000029368 | Alb | 5 | 90460897 | 90476602 | 0.37 | 3.91E-04 | 2.24E-02 |

**S3 Table. Down-regulated gene list in *Gprc6a^_KGKY-knockin^*** **mice.**

The complete list of down-regulated genes in livers from *Gprc6a^_KGKY-knockin^* mice compared to controls.

| **Ensembl Gene ID** | **Gene name** | **Chromosome** | **Gene start (bp)** | **Gene end (bp)** | **ky vs WT-log2FoldChange** | **ky vs WT-pvalue** | **ky vs WT-padj** |
| --- | --- | --- | --- | --- | --- | --- | --- |
| ENSMUSG00000030650 | Tmc5 | 7 | 118597297 | 118675086 | -10.50 | 3.35E-15 | 2.82E-12 |
| ENSMUSG00000072476 | Gm9008 | 6 | 76495432 | 76497784 | -8.72 | 1.57E-12 | 8.67E-10 |
| ENSMUSG00000030703 | Gdpd3 | 7 | 126766334 | 126775649 | -8.57 | 5.29E-29 | 9.40E-26 |
| ENSMUSG00000085666 | Gm9855 | 10 | 42054113 | 42055306 | -7.98 | 1.80E-13 | 1.20E-10 |
| ENSMUSG00000082329 | Gm14287 | 2 | 157528869 | 157528985 | -6.85 | 4.25E-07 | 7.55E-05 |
| ENSMUSG00000044976 | Wdr72 | 9 | 74110356 | 74283308 | -6.51 | 1.41E-07 | 2.92E-05 |
| ENSMUSG00000094497 | Gm8210 | 1 | 43189156 | 43189638 | -6.39 | 3.13E-19 | 4.55E-16 |
| ENSMUSG00000059751 | Rps3a3 | 13 | 108670603 | 108671397 | -5.96 | 2.20E-30 | 4.40E-27 |
| ENSMUSG00000040136 | Abcc8 | 7 | 46104523 | 46180033 | -5.53 | 2.89E-04 | 1.74E-02 |
| ENSMUSG00000023964 | Calcr | 6 | 3685680 | 3764714 | -5.39 | 1.25E-04 | 9.09E-03 |
| ENSMUSG00000056863 | Olfr702 | 7 | 106823489 | 106833893 | -4.98 | 4.78E-04 | 2.58E-02 |
| ENSMUSG00000025003 | Cyp2c39 | 19 | 39510862 | 39568529 | -4.69 | 4.80E-09 | 1.39E-06 |
| ENSMUSG00000058126 | Tpm3-rs7 | 14 | 113314608 | 113316754 | -4.66 | 5.19E-55 | 2.07E-51 |
| ENSMUSG00000079845 | Xlr4a | X | 73074345 | 73082478 | -4.50 | 2.14E-06 | 3.06E-04 |
| ENSMUSG00000082145 | Gm12312 | 11 | 70358138 | 70358781 | -4.50 | 1.77E-04 | 1.17E-02 |
| ENSMUSG00000059058 | Tma7-ps | 1 | 171711088 | 171711282 | -4.32 | 5.95E-47 | 1.90E-43 |
| ENSMUSG00000043773 | 1700048O20Rik | 9 | 121937223 | 121947016 | -4.23 | 6.66E-05 | 5.41E-03 |
| ENSMUSG00000010066 | Cacna2d2 | 9 | 107399612 | 107529343 | -4.10 | 1.03E-03 | 4.69E-02 |
| ENSMUSG00000086962 | Gm12248 | 11 | 58063552 | 58093134 | -3.87 | 1.33E-05 | 1.48E-03 |
| ENSMUSG00000107092 | Gm7993 | 5 | 95974240 | 95975623 | -3.84 | 9.49E-04 | 4.48E-02 |
| ENSMUSG00000097662 | Gm2093 | 15 | 3996039 | 4015858 | -3.70 | 1.64E-09 | 5.68E-07 |
| ENSMUSG00000080893 | Gm15920 | 5 | 130188395 | 130188796 | -3.48 | 8.11E-24 | 1.30E-20 |
| ENSMUSG00000103953 | Gm29718 | 1 | 136793034 | 136793510 | -3.35 | 6.29E-13 | 3.87E-10 |
| ENSMUSG00000076612 | Ighg2c | 12 | 113285325 | 113288932 | -3.32 | 2.08E-12 | 1.11E-09 |
| ENSMUSG00000029752 | Asns | 6 | 7675169 | 7693254 | -3.26 | 1.74E-06 | 2.56E-04 |
| ENSMUSG00000028359 | Orm3 | 4 | 63356162 | 63359511 | -3.25 | 4.52E-11 | 1.90E-08 |
| ENSMUSG00000062611 | Rps3a2 | 14 | 88123017 | 88123808 | -3.24 | 2.58E-46 | 6.87E-43 |
| ENSMUSG00000023087 | Noct | 3 | 51224447 | 51251644 | -3.22 | 2.28E-78 | 3.65E-74 |
| ENSMUSG00000050550 | Gm11868 | 4 | 18844202 | 18845679 | -3.17 | 1.39E-14 | 1.01E-11 |
| ENSMUSG00000063953 | Amd2 | 10 | 35708675 | 35711892 | -3.11 | 5.92E-18 | 6.77E-15 |
| ENSMUSG00000020185 | E2f7 | 10 | 110745439 | 110787384 | -3.09 | 6.17E-06 | 7.82E-04 |
| ENSMUSG00000084349 | Rpl3-ps1 | X | 13202571 | 13203780 | -3.06 | 1.46E-63 | 7.78E-60 |
| ENSMUSG00000093916 | Gm379 | X | 108664004 | 108664891 | -2.89 | 8.88E-04 | 4.26E-02 |
| ENSMUSG00000074280 | Gm6166 | 9 | 57483963 | 57484371 | -2.82 | 1.93E-09 | 6.43E-07 |
| ENSMUSG00000036744 | Olfr701 | 7 | 106814129 | 106821472 | -2.81 | 8.90E-04 | 4.26E-02 |
| ENSMUSG00000091476 | Catspere2 | 1 | 177983423 | 178172704 | -2.75 | 4.24E-07 | 7.55E-05 |
| ENSMUSG00000067608 | Pcna-ps2 | 19 | 9283238 | 9284494 | -2.58 | 7.79E-11 | 3.11E-08 |
| ENSMUSG00000028008 | Asic5 | 3 | 81982290 | 82021233 | -2.58 | 1.03E-37 | 2.36E-34 |
| ENSMUSG00000023968 | Crip3 | 17 | 46428926 | 46431776 | -2.49 | 7.67E-04 | 3.79E-02 |
| ENSMUSG00000097451 | Rian | 12 | 109603940 | 109661716 | -2.40 | 3.23E-04 | 1.92E-02 |
| ENSMUSG00000000739 | Sult5a1 | 8 | 123140192 | 123158315 | -2.40 | 4.75E-05 | 4.08E-03 |
| ENSMUSG00000068877 | Selenbp2 | 3 | 94693556 | 94704413 | -2.27 | 8.79E-06 | 1.04E-03 |
| ENSMUSG00000061540 | Orm2 | 4 | 63362449 | 63365878 | -2.25 | 2.81E-04 | 1.71E-02 |
| ENSMUSG00000083833 | Gm13841 | 5 | 115801677 | 115802159 | -2.15 | 7.40E-09 | 2.11E-06 |
| ENSMUSG00000053702 | Nebl | 2 | 17343909 | 17731464 | -2.11 | 7.82E-08 | 1.81E-05 |
| ENSMUSG00000067144 | Slc22a7 | 17 | 46432183 | 46438457 | -2.08 | 4.89E-18 | 6.02E-15 |
| ENSMUSG00000067149 | Jchain | 5 | 88519809 | 88527891 | -2.06 | 2.03E-08 | 5.43E-06 |
| ENSMUSG00000095385 | D630033O11Rik | 9 | 43243599 | 43280076 | -2.05 | 1.30E-06 | 2.01E-04 |
| ENSMUSG00000018648 | Dusp14 | 11 | 84048041 | 84069261 | -2.04 | 5.46E-04 | 2.84E-02 |
| ENSMUSG00000066705 | Fxyd6 | 9 | 45370185 | 45396159 | -2.02 | 1.18E-11 | 5.82E-09 |
| ENSMUSG00000001642 | Akr1b3 | 6 | 34302434 | 34317478 | -1.92 | 3.94E-04 | 2.25E-02 |
| ENSMUSG00000081223 | Gm12247 | 11 | 58097104 | 58097773 | -1.91 | 9.44E-07 | 1.51E-04 |
| ENSMUSG00000032080 | Apoa4 | 9 | 46240696 | 46243459 | -1.85 | 4.25E-16 | 4.25E-13 |
| ENSMUSG00000043300 | B3galnt1 | 3 | 69574158 | 69598960 | -1.85 | 7.00E-04 | 3.50E-02 |
| ENSMUSG00000049313 | Sorl1 | 9 | 41964720 | 42124297 | -1.82 | 2.36E-10 | 8.98E-08 |
| ENSMUSG00000029380 | Cxcl1 | 5 | 90891241 | 90893115 | -1.81 | 1.69E-07 | 3.38E-05 |
| ENSMUSG00000040483 | Xaf1 | 11 | 72301629 | 72313733 | -1.77 | 1.70E-09 | 5.79E-07 |
| ENSMUSG00000043753 | Dmrta1 | 4 | 89679436 | 89694772 | -1.77 | 4.35E-05 | 3.81E-03 |
| ENSMUSG00000024697 | Gna14 | 19 | 16435667 | 16610818 | -1.70 | 2.07E-08 | 5.43E-06 |
| ENSMUSG00000072568 | Fam84b | 15 | 60818994 | 60853778 | -1.65 | 3.99E-08 | 1.03E-05 |
| ENSMUSG00000076609 | Igkc | 6 | 70726435 | 70726966 | -1.64 | 8.94E-04 | 4.27E-02 |
| ENSMUSG00000054074 | Skida1 | 2 | 18040676 | 18049051 | -1.61 | 4.60E-05 | 3.98E-03 |
| ENSMUSG00000094156 | Sult2a7 | 7 | 14465051 | 14494230 | -1.53 | 6.70E-07 | 1.13E-04 |
| ENSMUSG00000031565 | Fgfr1 | 8 | 25513654 | 25575718 | -1.49 | 1.20E-11 | 5.82E-09 |
| ENSMUSG00000046380 | Jrk | 15 | 74702301 | 74709535 | -1.44 | 5.09E-04 | 2.68E-02 |
| ENSMUSG00000003469 | Phyhip | 14 | 70457476 | 70468832 | -1.41 | 1.01E-03 | 4.66E-02 |
| ENSMUSG00000038984 | Tspyl5 | 15 | 33683875 | 33687884 | -1.41 | 1.87E-05 | 1.94E-03 |
| ENSMUSG00000021508 | Cxcl14 | 13 | 56288643 | 56296551 | -1.41 | 1.99E-04 | 1.28E-02 |
| ENSMUSG00000102375 | A930036K24Rik | 9 | 107680082 | 107681281 | -1.40 | 4.57E-05 | 3.97E-03 |
| ENSMUSG00000085006 | BC021767 | 3 | 94661830 | 94670696 | -1.39 | 8.72E-05 | 6.67E-03 |
| ENSMUSG00000081058 | Hist2h3c2 | 3 | 96238108 | 96239127 | -1.37 | 1.21E-04 | 8.86E-03 |
| ENSMUSG00000022415 | Syngr1 | 15 | 80091334 | 80119501 | -1.37 | 7.89E-04 | 3.87E-02 |
| ENSMUSG00000031441 | Atp11a | 8 | 12757014 | 12868728 | -1.34 | 1.32E-07 | 2.79E-05 |
| ENSMUSG00000001761 | Smo | 6 | 29735503 | 29761365 | -1.31 | 3.46E-13 | 2.21E-10 |
| ENSMUSG00000039519 | Cyp7b1 | 3 | 18071950 | 18243338 | -1.26 | 4.79E-08 | 1.20E-05 |
| ENSMUSG00000023031 | Cela1 | 15 | 100674421 | 100687921 | -1.24 | 1.31E-07 | 2.78E-05 |
| ENSMUSG00000104445 | Rhbg | 3 | 88242874 | 88254709 | -1.24 | 1.12E-05 | 1.28E-03 |
| ENSMUSG00000060613 | Cyp2c70 | 19 | 40153353 | 40187333 | -1.19 | 6.43E-19 | 8.57E-16 |
| ENSMUSG00000068101 | Cenpm | 15 | 82233779 | 82244748 | -1.17 | 1.68E-04 | 1.13E-02 |
| ENSMUSG00000067279 | Ppp1r3c | 19 | 36731737 | 36736653 | -1.16 | 5.74E-10 | 2.13E-07 |
| ENSMUSG00000039347 | Atp6v0e2 | 6 | 48537615 | 48541801 | -1.15 | 6.71E-04 | 3.42E-02 |
| ENSMUSG00000054855 | Rnd1 | 15 | 98663421 | 98677461 | -1.13 | 1.67E-07 | 3.37E-05 |
| ENSMUSG00000024427 | Spry4 | 18 | 38586268 | 38601268 | -1.10 | 2.83E-05 | 2.78E-03 |
| ENSMUSG00000000567 | Sox9 | 11 | 112782224 | 112787760 | -1.08 | 1.64E-04 | 1.11E-02 |
| ENSMUSG00000026442 | Nfasc | 1 | 132564690 | 132741797 | -1.06 | 4.37E-04 | 2.44E-02 |
| ENSMUSG00000038147 | Cd84 | 1 | 171839697 | 171890718 | -1.05 | 2.06E-04 | 1.32E-02 |
| ENSMUSG00000045087 | S1pr5 | 9 | 21242912 | 21248443 | -1.02 | 1.22E-05 | 1.38E-03 |
| ENSMUSG00000027175 | Tcp11l1 | 2 | 104657288 | 104712169 | -1.01 | 1.23E-07 | 2.66E-05 |
| ENSMUSG00000018008 | Cyth4 | 15 | 78597047 | 78622019 | -0.98 | 5.26E-04 | 2.75E-02 |
| ENSMUSG00000027556 | Car1 | 3 | 14766216 | 14808368 | -0.96 | 5.05E-05 | 4.30E-03 |
| ENSMUSG00000050663 | Trhde | 10 | 114398823 | 114802307 | -0.95 | 3.77E-06 | 5.20E-04 |
| ENSMUSG00000035561 | Aldh1b1 | 4 | 45799022 | 45804604 | -0.95 | 3.01E-09 | 9.64E-07 |
| ENSMUSG00000002396 | Ocel1 | 8 | 71371298 | 71379361 | -0.94 | 2.59E-04 | 1.60E-02 |
| ENSMUSG00000046688 | Tifa | 3 | 127789805 | 127832164 | -0.92 | 5.03E-06 | 6.76E-04 |
| ENSMUSG00000030587 | 2200002D01Rik | 7 | 29246561 | 29248466 | -0.90 | 7.10E-06 | 8.60E-04 |
| ENSMUSG00000026117 | Zap70 | 1 | 36761798 | 36782818 | -0.88 | 3.63E-05 | 3.30E-03 |
| ENSMUSG00000049907 | Rasl11b | 5 | 74195286 | 74199481 | -0.88 | 1.01E-03 | 4.66E-02 |
| ENSMUSG00000039621 | Prex1 | 2 | 166566342 | 166713832 | -0.88 | 2.43E-04 | 1.51E-02 |
| ENSMUSG00000020623 | Map2k6 | 11 | 110399122 | 110525522 | -0.87 | 7.80E-04 | 3.84E-02 |
| ENSMUSG00000025044 | Msr1 | 8 | 39581685 | 39642673 | -0.87 | 1.36E-04 | 9.76E-03 |
| ENSMUSG00000025498 | Irf7 | 7 | 141262706 | 141266481 | -0.86 | 5.95E-05 | 4.90E-03 |
| ENSMUSG00000071669 | Snx29 | 16 | 11322908 | 11755472 | -0.86 | 5.92E-06 | 7.63E-04 |
| ENSMUSG00000061906 | Ugt2b38 | 5 | 87409942 | 87424203 | -0.85 | 4.99E-05 | 4.27E-03 |
| ENSMUSG00000041729 | Coro2b | 9 | 62419492 | 62537044 | -0.85 | 6.74E-04 | 3.42E-02 |
| ENSMUSG00000057068 | Fam47e | 5 | 92555069 | 92591279 | -0.84 | 1.12E-04 | 8.38E-03 |
| ENSMUSG00000020427 | Igfbp3 | 11 | 7206086 | 7213923 | -0.83 | 1.19E-04 | 8.84E-03 |
| ENSMUSG00000025964 | Adam23 | 1 | 63445891 | 63596276 | -0.82 | 3.71E-05 | 3.35E-03 |
| ENSMUSG00000029534 | St7 | 6 | 17692933 | 17943025 | -0.82 | 2.89E-04 | 1.74E-02 |
| ENSMUSG00000015854 | Cd5l | 3 | 87357881 | 87371073 | -0.81 | 4.28E-04 | 2.41E-02 |
| ENSMUSG00000021376 | Tpmt | 13 | 47022482 | 47044737 | -0.80 | 5.69E-05 | 4.71E-03 |
| ENSMUSG00000068246 | Apol9b | 15 | 77729039 | 77736382 | -0.79 | 4.46E-04 | 2.46E-02 |
| ENSMUSG00000025880 | Smad7 | 18 | 75367529 | 75395935 | -0.78 | 6.51E-04 | 3.34E-02 |
| ENSMUSG00000027199 | Gatm | 2 | 122594467 | 122611303 | -0.77 | 4.26E-04 | 2.41E-02 |
| ENSMUSG00000028195 | Cyr61 | 3 | 145646976 | 145649981 | -0.76 | 3.77E-06 | 5.20E-04 |
| ENSMUSG00000053716 | Dusp7 | 9 | 106368632 | 106375724 | -0.75 | 7.37E-05 | 5.81E-03 |
| ENSMUSG00000025200 | Cwf19l1 | 19 | 44108644 | 44135876 | -0.75 | 2.23E-07 | 4.30E-05 |
| ENSMUSG00000009772 | Nuak2 | 1 | 132316126 | 132333488 | -0.74 | 5.21E-05 | 4.37E-03 |
| ENSMUSG00000003500 | Impdh1 | 6 | 29200434 | 29216364 | -0.74 | 2.95E-04 | 1.76E-02 |
| ENSMUSG00000025350 | Rdh5 | 10 | 128913593 | 128922888 | -0.74 | 8.08E-05 | 6.21E-03 |
| ENSMUSG00000019960 | Dusp6 | 10 | 99263231 | 99267489 | -0.73 | 4.63E-04 | 2.53E-02 |
| ENSMUSG00000044206 | Vsig4 | X | 96247203 | 96293438 | -0.73 | 1.02E-03 | 4.68E-02 |
| ENSMUSG00000039196 | Orm1 | 4 | 63344560 | 63348163 | -0.73 | 1.73E-06 | 2.56E-04 |
| ENSMUSG00000039246 | Lyplal1 | 1 | 186087731 | 186117310 | -0.72 | 1.42E-05 | 1.55E-03 |
| ENSMUSG00000032263 | Bckdhb | 9 | 83925145 | 84124240 | -0.72 | 3.57E-11 | 1.58E-08 |
| ENSMUSG00000014542 | Clec4f | 6 | 83644542 | 83656187 | -0.70 | 7.60E-05 | 5.93E-03 |
| ENSMUSG00000037621 | Atoh8 | 6 | 72206177 | 72235577 | -0.70 | 7.13E-04 | 3.55E-02 |
| ENSMUSG00000045854 | Lyrm2 | 4 | 32800253 | 32801559 | -0.69 | 1.69E-04 | 1.13E-02 |
| ENSMUSG00000006522 | Itih3 | 14 | 30908572 | 30923760 | -0.69 | 1.30E-04 | 9.43E-03 |
| ENSMUSG00000030091 | Nup210 | 6 | 91013068 | 91116829 | -0.69 | 1.90E-04 | 1.24E-02 |
| ENSMUSG00000031431 | Tsc22d3 | X | 140539528 | 140600659 | -0.69 | 8.64E-04 | 4.19E-02 |
| ENSMUSG00000040658 | Dnph1 | 17 | 46496711 | 46499624 | -0.69 | 4.72E-04 | 2.57E-02 |
| ENSMUSG00000067225 | Cyp2c54 | 19 | 40037941 | 40073811 | -0.68 | 4.99E-04 | 2.66E-02 |
| ENSMUSG00000037826 | Ppm1k | 6 | 57506502 | 57535468 | -0.67 | 1.54E-05 | 1.64E-03 |
| ENSMUSG00000055652 | Klhl25 | 7 | 75848310 | 75874131 | -0.67 | 1.45E-04 | 1.02E-02 |
| ENSMUSG00000038005 | Hpf1 | 8 | 60890418 | 60908671 | -0.67 | 3.35E-04 | 1.98E-02 |
| ENSMUSG00000074768 | Bhmt | 13 | 93616675 | 93637961 | -0.66 | 9.75E-05 | 7.43E-03 |
| ENSMUSG00000032575 | Manf | 9 | 106838312 | 106891979 | -0.66 | 4.85E-04 | 2.60E-02 |
| ENSMUSG00000018800 | Abca5 | 11 | 110269369 | 110337716 | -0.66 | 9.52E-04 | 4.48E-02 |
| ENSMUSG00000020108 | Ddit4 | 10 | 59949669 | 59951834 | -0.66 | 8.58E-04 | 4.17E-02 |
| ENSMUSG00000036867 | Smad6 | 9 | 63953076 | 64022059 | -0.64 | 4.60E-04 | 2.53E-02 |
| ENSMUSG00000040699 | Limd2 | 11 | 106156256 | 106160860 | -0.63 | 1.53E-05 | 1.64E-03 |
| ENSMUSG00000022304 | Dpys | 15 | 39768487 | 39857470 | -0.62 | 1.57E-07 | 3.22E-05 |
| ENSMUSG00000022615 | Tymp | 15 | 89371931 | 89377039 | -0.62 | 2.91E-05 | 2.83E-03 |
| ENSMUSG00000057228 | Aadat | 8 | 60505932 | 60545677 | -0.62 | 3.29E-04 | 1.95E-02 |
| ENSMUSG00000022149 | C9 | 15 | 6445327 | 6498751 | -0.60 | 1.49E-04 | 1.04E-02 |
| ENSMUSG00000025190 | Got1 | 19 | 43499752 | 43524605 | -0.59 | 1.42E-04 | 1.01E-02 |
| ENSMUSG00000034947 | Tmem106a | 11 | 101582242 | 101591788 | -0.58 | 9.66E-04 | 4.51E-02 |
| ENSMUSG00000022498 | Txndc11 | 16 | 11074911 | 11134650 | -0.56 | 1.63E-04 | 1.11E-02 |
| ENSMUSG00000051319 | 1500011K16Rik | 2 | 127791388 | 127792488 | -0.55 | 1.11E-03 | 4.96E-02 |
| ENSMUSG00000040413 | Timd2 | 11 | 46668960 | 46707061 | -0.55 | 8.95E-07 | 1.45E-04 |
| ENSMUSG00000027983 | Cyp2u1 | 3 | 131288441 | 131303227 | -0.55 | 9.77E-04 | 4.53E-02 |
| ENSMUSG00000055312 | Them7 | 2 | 105224320 | 105379796 | -0.54 | 4.25E-04 | 2.41E-02 |
| ENSMUSG00000040033 | Stat2 | 10 | 128270559 | 128292849 | -0.54 | 1.75E-05 | 1.85E-03 |
| ENSMUSG00000029625 | Cpsf4 | 5 | 145167213 | 145182041 | -0.53 | 1.57E-04 | 1.09E-02 |
| ENSMUSG00000036083 | Slc17a3 | 13 | 23839434 | 23860716 | -0.52 | 1.20E-04 | 8.85E-03 |
| ENSMUSG00000030895 | Hpx | 7 | 105591613 | 105600137 | -0.51 | 1.03E-03 | 4.69E-02 |
| ENSMUSG00000003355 | Fkbp11 | 15 | 98724366 | 98728198 | -0.49 | 9.10E-04 | 4.32E-02 |
| ENSMUSG00000042102 | Dmgdh | 13 | 93674433 | 93752833 | -0.49 | 6.66E-05 | 5.41E-03 |
| ENSMUSG00000022337 | Emc2 | 15 | 43477229 | 43527763 | -0.44 | 3.76E-04 | 2.16E-02 |
| ENSMUSG00000019433 | Gipc1 | 8 | 83652677 | 83664694 | -0.43 | 5.09E-04 | 2.68E-02 |
| ENSMUSG00000051716 | Apon | 10 | 128254096 | 128255896 | -0.42 | 3.08E-05 | 2.91E-03 |
| ENSMUSG00000026272 | Agxt | 1 | 93135240 | 93145421 | -0.39 | 6.80E-04 | 3.43E-02 |

**The image of full-length gel for Figure 4c in main text.**
